# Visible-Light-Induced Carbonylative Synthesis of Aliphatic Thioesters

**DOI:** 10.1021/acs.orglett.6c00753

**Published:** 2026-03-26

**Authors:** Ren-Guan Miao, Ru-Han A, Xiao-Feng Wu

**Affiliations:** † Dalian National Laboratory for Clean Energy, Dalian Institute of Chemical Physics, Chinese Academy of Sciences, Dalian, Liaoning 116023, China; ‡ Leibniz-Institut für Katalyse e.V., 18059 Rostock, Germany

## Abstract

This study presents a visible-light-induced metal-free catalytic carbonylation method for the synthesis of alkyl thioesters. Utilizing Hantzsch esters as alkyl radical precursors, the method selectively captures aryl sulfonates from thiosulfonates via acyl radical intermediates in the presence of carbon monoxide, efficiently constructing alkyl thioesters. The reaction system is characterized by its mildness, which leads to the production of a range of thioester products in high yields with excellent functional group tolerance. Thiosulfonates, classified as bifunctional reagents, serve a dual role in this process. First, they function as a source of sulfur. Second, the arylsulfonyl radicals generate act as endogenous oxidants, thereby maintaining the reaction cycle.

## Graphical abstract

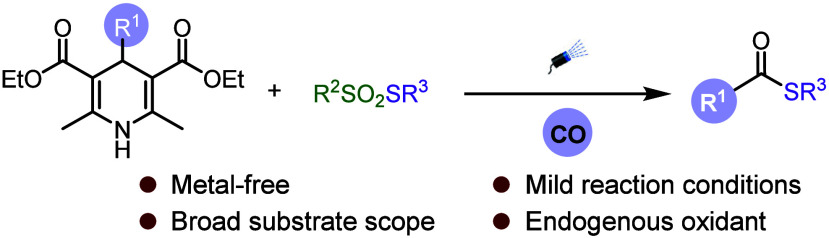

Thioester compounds are prevalent in drug molecules and bioactive substances and are also key intermediates in the field of synthetic chemistry. The primary rationale for their focus pertains to the distinctive biological characteristics inherent in the thioester functional group.^1^ These compounds demonstrate notable stability in ambient air; in comparison to other esters, thioesters exhibit a diminished orbital conjugation overlap effect. This structural characteristic renders them excellent acyl donors, allowing for the preparation of various important organic compounds, such as ketones, aldehydes, esters, amides, and acylsilanes through transformation reactions. In addition, thioesters are significant as raw materials in a number of different areas. These include the synthesis of proteins, functional materials, food additives and antibiotics.^2,3^ Consequently, the development of novel, efficient, and ecofriendly thioester synthesis methods is of significant research and application value. The preparation of thioesters by conventional methods principally relies upon condensation reactions between thiols and carboxylic acids or acid chlorides.^4,5^ Thiols, a commonly used source of sulfur, are characterized by a strong and irritating odor. Furthermore, they are susceptible to catalyst poisoning and deactivation. Consequently, the utilization of non-toxic and efficient sulfur sources in thioester synthesis strategies is of significant practical importance (Scheme 1A).

**Scheme 1:**
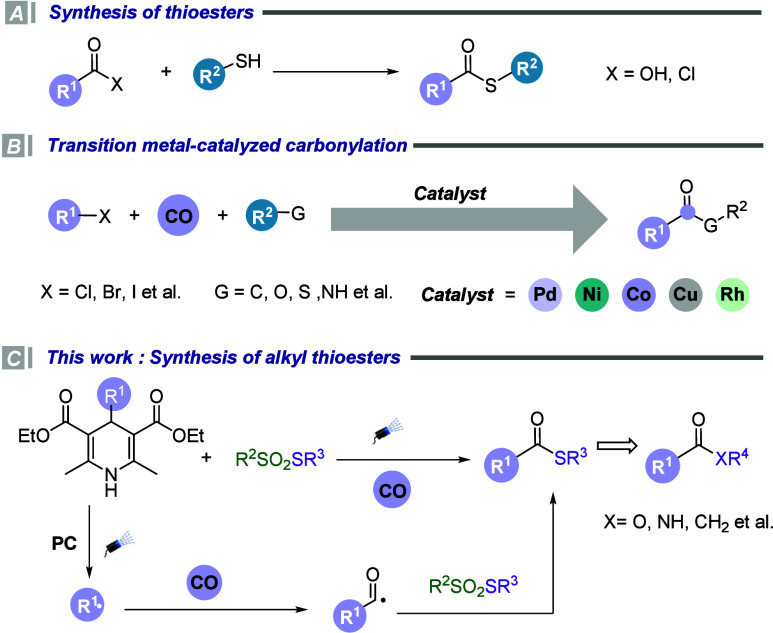
Carbonylative Synthesis of Aliphatic Thioesters

The carbonyl group is a significant functional group within the domain of organic compounds. The structural diversity of these compounds gives rise to their unique biological activity and catalytic performance, thus positioning them as being of significant importance in both fundamental research and industrial-scale organic synthesis.^6–11^ According to the relevant statistics, from 2015 to 2020, approximately 80% of the small-molecule drugs approved for marketing by the U.S. Food and Drug Administration (FDA) contained carbonyl fragments in their molecular structures. These data fully confirm the significant application value of carbonyl groups in the field of drug research and development.^12^ In the research and application of carbonylation reactions, carbon monoxide (CO) is utilized as a chemical raw material, which is both inexpensive and readily available. This material combines the advantages of mature preparation technology and easy large-scale production. It functions as a pivotal conduit, integrating fundamental chemical raw materials with high-value-added complex carbonyl compounds, and its utilization is extensively prevalent in both the academic and industrial domains. Following the initial report on the direct carbonylation of aryl halides catalyzed by palladium for the preparation of carbonyl compounds in 1974,^13^ research on transition-metal-catalyzed carbonylation reactions has undergone rapid development. The employment of transition metal catalysts (Pd, Rh, Co, Ni, and Cu) enables the efficient coupling of catalysts with diverse substrates, including olefins, alcohols, amines, and halogenated hydrocarbons. This process facilitates the introduction of carbonyl functional groups with high selectivity and efficiency. This synthesis represents a significant theoretical and practical research achievement, encompassing a range of carbonyl compounds, including aldehydes, carboxylic acids, amides, ketones, esters, and thioesters (Scheme 1B).^14–19^

Radical carbonylation chemistry, which has been shown to efficiently prepare carbonylation products under metal-free conditions, serves as an attractive complementary approach to transition-metal-catalyzed strategies.^20–26^ The radical coupling reactions mediated by photocatalytic single-electron transfer (SET) provide a new, environmentally friendly, and atomically efficient pathway for the construction of complex molecular skeletons. In recent years, photocatalyzed SET-mediated carbonylation of CO has become one of the main research focuses in academia. Hantzsch esters, with their unique light absorption properties and electron transfer characteristics, can serve as alkyl radical precursors.^27^ Based on this, it is proposed that using Hantzsch esters as alkyl radical precursors to prepare thioester compounds through a visible-light-induced metal-free carbonylation reaction pathway would be a feasible and innovative synthesis route. In addition, thiosulfonates, as a class of excellent bifunctional reagents, are ideal choices for this reaction system. The favorable leaving group nature of the phenylsulfonyl group, which is susceptible to nucleophilic attack, enables its function as both a sulfur source and a catalyst for the oxidation and regeneration of the photosensitizer during the reaction process, thereby ensuring a continuous catalytic cycle (Scheme 1C).

Based on the aforementioned reaction design approach, this study utilized diethyl 4-cyclohexyl-2,6-dimethyl-1,4-dihydropyridine-3,5-dicarboxylate (**1a**) and *S*-(4-methoxyphenyl)­benzenesulfonothioate (**2a**) as template substrates to conduct screening and optimization of reaction conditions. The initial reaction was carried out using Ir­[dF­(CF_3_)­ppy]_2_(dtbbpy)­PF_6_ as the photocatalyst, under irradiation from 15 W blue LEDs (456 nm), in THF at room temperature under 60 bar of CO atmosphere, initially achieving a target product yield of 40% (Table 1, entry 1). Subsequently, a systematic screening of reaction solvents was conducted, examining the impact of various solvents, such as DMAc, MeCN, and toluene, on the reaction yield. The experimental results indicated that none of these solvents improved the reaction yield. Based on this, THF was determined as the optimal solvent for this reaction (Table 1, entries 2–5). Further optimization of the substrate ratio was carried out by adjusting the feeding ratio of **1a** to **2a**, resulting in an increased yield of the target product to 61% (Table 1, entry 6). Based on this, the types of photocatalysts were screened and the catalytic performance of different types of photocatalysts was investigated. It was found that, when ruthenium (Ru) compounds were used as photocatalysts, the reaction yield significantly decreased, whereas when 4CzIPN was used as the photocatalyst, the yield of the target thioester product could be increased to 76% (Table 1, entries 7–11). By further adjustment of the solvent dosage, it was discovered that, when the amount of THF was 2.5 mL, the yield of the target product could be further increased to 85% (Table 1, entry 11). Additionally, an attempt was made to reduce the CO atmosphere pressure to 40 bar, resulting in a decrease in the reaction yield to 70% (Table 1, entry 12). Notably, a low yield of **3q** (21%) was obtained if we stopped the reaction after 10 h. Notably, similar results (25%) were obtained if we stopped light irradiation after 10 h and then continued the reaction for another 14 h under dark. In summary, through systematic screening and optimization of key reaction parameters, such as the reaction solvent, substrate ratio, photocatalyst type, solvent dosage, and CO pressure, the optimal reaction conditions for this reaction were finally determined as follows: 4CzIPN (1 mol %), THF, CO (60 bar), rt, and 15 W blue LEDs.

**Table 1:**
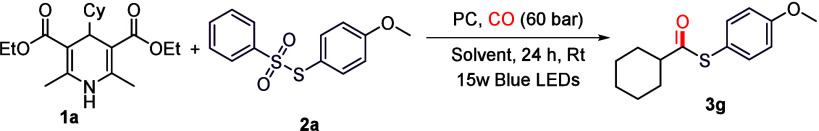
Optimization of the Reaction Conditions^a^

entry	photocatalyst	solvent	yield (%)
1	Ir[dF(CF_3_)ppy]_2_(dtbbpy)PF_6_	THF	40
2	Ir[dF(CF_3_)ppy]_2_(dtbbpy)PF_6_	DMAc	13
3	Ir[dF(CF_3_)ppy]_2_(dtbbpy)PF_6_	MeCN	16
4	Ir[dF(CF_3_)ppy]_2_(dtbbpy)PF_6_	toluene	31
5	Ir[dF(CF_3_)ppy]_2_(dtbbpy)PF_6_	DMF	17
6^b^	Ir[dF(CF_3_)ppy]_2_(dtbbpy)PF_6_	THF	61
7^b^	4CzIPN	THF	76
8^b^	*fac*-(Irppy)_3_	THF	44
9^b^	Ru(bpy)_3_Cl_2_·6H_2_O	THF	16
10^b^	Ir(ppy)_2_(dtbbpy)PF_6_	THF	61
11^b^ ^,^ ^c^	4CzIPN	THF	85
12^b^ ^,^ ^c^ ^,^ ^d^	4CzIPN	THF	70

a Reaction conditions: **1a** (0.1 mmol), **2a** (0.15 mmol), Ir­[dF­(CF_3_)­ppy]_2_(dtbbpy)­PF_6_ (1 mol %), THF (1 mL), CO (60 bar), rt, 24 h, and 15 W blue LEDs, with isolated yields.

b **1a** (0.25 mmol) and **2a** (0.1 mmol).

c THF (2.5 mL).

d CO (40 bar).

Following the determination of the optimal reaction conditions, the generality of this carbonylation method toward substrates with different substituents was investigated. In the course of our investigation, we examined the reactions between a range of Hantzsch esters and thiosulfonates under standard reaction conditions (Scheme 2). First, utilizing 4-cyclohexyl-2,6-dimethyl-1,4-dihydropyridine-3,5-dicarboxylic acid diethyl ester (**1a**) as the template substrate, a series of reactions were conducted with various thiosulfonates bearing electron-donating groups (methyl, isopropyl, *tert*-butyl, and methoxy) on the benzene ring. These reactions consistently yielded favorable outcomes with yields ranging from 70 to 96% for the desired products **3a**–**3h**. It is noteworthy that thiosulfonates with methyl groups at the *meta* and *para* positions of the benzene ring still yielded high yields. This finding suggests that an increase in steric hindrance is beneficial for enhancing the reaction efficiency of **3b**–**3d**. In the ensuing investigation, a range of thiosulfonates bearing electron-withdrawing groups (fluorine, chlorine, bromine, and trifluoromethyl) on the benzene ring were examined, resulting in the successful synthesis of target products **3i**–**3l**, with yields ranging from 50 to 94%. It is noteworthy that the reaction proceeded in a satisfactory manner when the halogen substituent was located at the *para* position of the benzene ring. This is in contrast to the typical adverse effect of such a substituent on traditional transition-metal-catalyzed carbonylation reactions for the synthesis of thioesters. Subsequently, reaction studies were conducted on heterocyclic-substituted thiosulfonates, which resulted in the detection of a minimal amount of the thioester product **3m**. The reaction with furan and pyridine derivatives failed as well. In a similar manner, alkyl-substituted thiosulfonates were found to be undetectable (**3n** and **3o**), with only trace amounts of the target product being detected. However, utilizing *S*-phenyl ethanesulfonyl thioate as the substrate, thioester **3a** was successfully obtained with a yield of 68%, thereby demonstrating the remarkable compatibility of the sulfonic acid group of arylsulfonyl thioates, irrespective of alkyl or aryl substitution, with this particular reaction system. In addition, we also investigated various substituted Hantzsch ester substrates, including isopropyl-, cyclopentyl-, and pyranyl-substituted Hantzsch esters, and the reactions all yielded the corresponding products **3p**–**3r** in good yields. When Hantzsch esters containing internal olefin structures were used in the reaction, target products **3r**–**3t** were also obtained in good yields. Finally, we tried different substituted aryl sulfonate substrates, but no target product was detected in the reaction system.

**Scheme 2:**
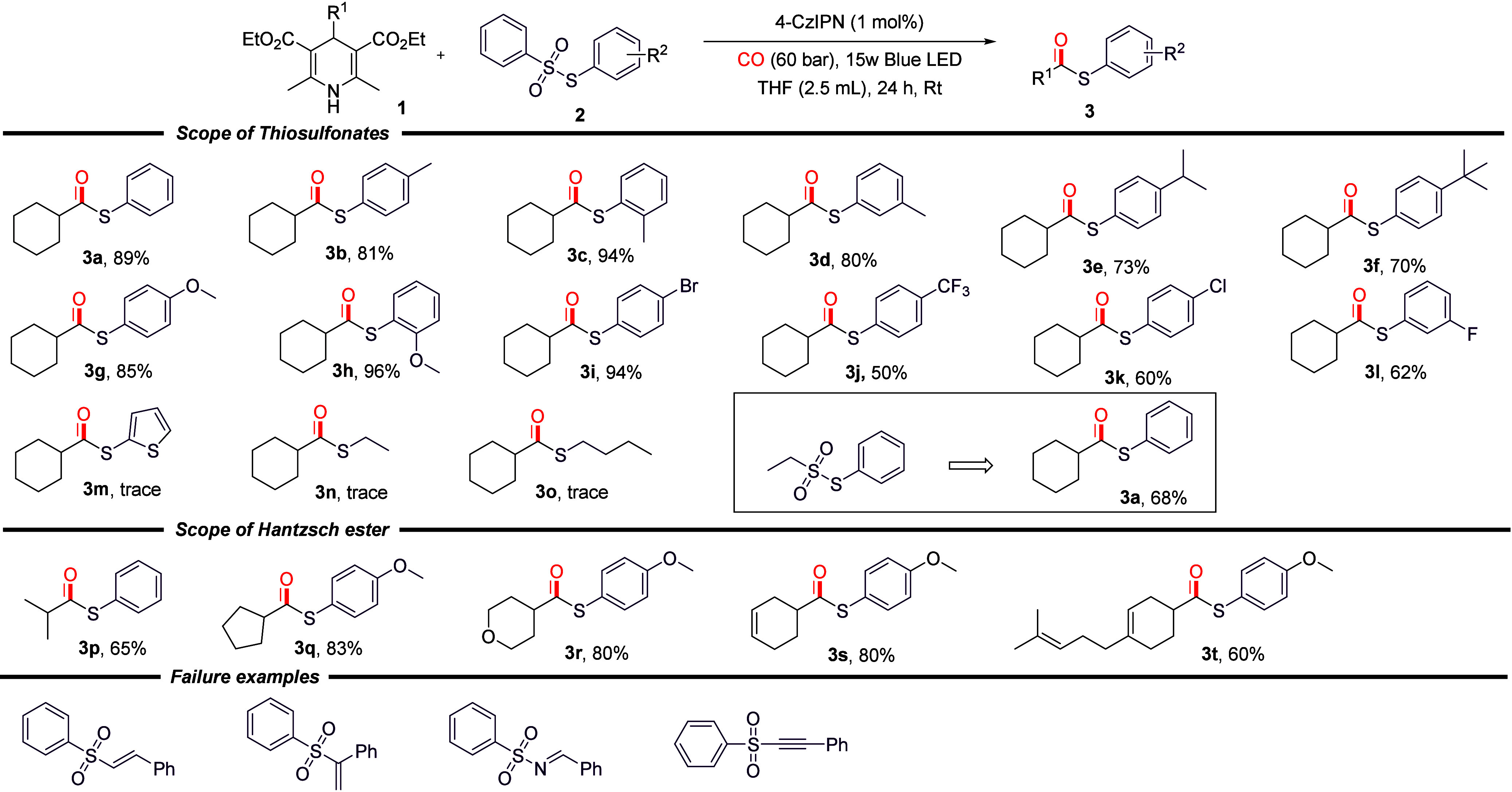
Substate Scope^a^

a Reaction conditions: **1a** (0.25 mmol), **2a** (0.1 mmol), 4CzIPN (1 mol %), THF (2.5 mL), CO (60 bar), rt, 24 h, and 15 W blue LEDs, with isolated yields.

To further investigate the mechanism of this reaction, we designed and conducted a series of control experiments. The relevant experimental results are detailed (Scheme 3). First, under standard reaction conditions, 2,2,6,6-Tetramethylpiperidine-1-oxyl radical (TEMPO) was added to verify whether the reaction proceeds through a radical reaction pathway. The experimental results showed that no target product was detected in the system, while related intermediate products were detected by HRMS. Subsequently, under the same standard conditions, the above experiment was repeated using 2,6-di-*tert*-butyl-4-methylphenol (BHT), and again, no target product was detected. Under standard conditions, the above experiment was repeated using ethene-1,1-diyldibenzene and related intermediates were detected. The results of the three sets of control experiments collectively indicate that this reaction most likely follows a radical reaction pathway. In addition, we also attempted to scale up the substrate amount to 1 mmol for reaction verification and finally obtained the target product in a yield of 70%. Meanwhile, to expand the application value of the product, we further explored the transformation reaction of the product and developed a nickel-catalyzed cross-coupling reaction using iodobenzene and thioester **3a** as raw materials for the synthesis of sulfides. Finally, corresponding sulfide product **4a** was successfully obtained in a yield of 80%.

**Scheme 3:**
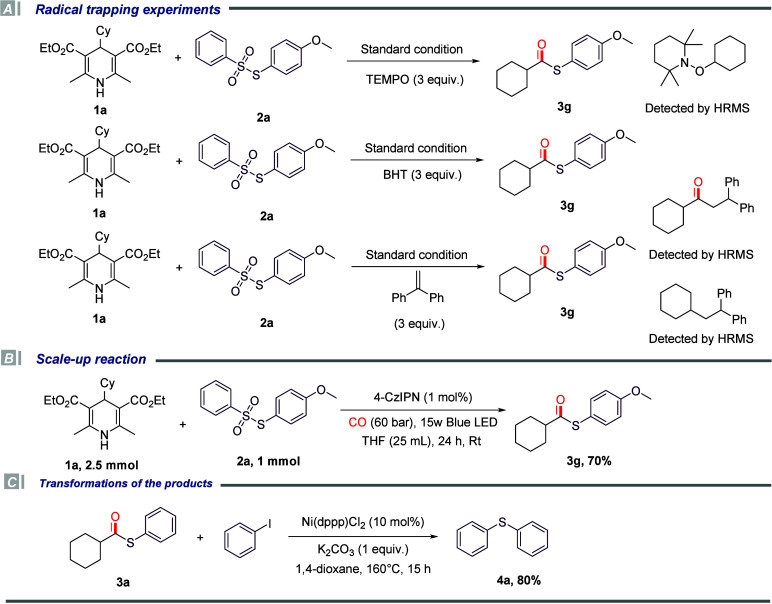
Reaction Control Experiments, Scale-Up, and Synthetic Applications

Based on the experimental results mentioned above and the reported relevant literature, we propose a possible reaction mechanism for the carbonylation reaction (Scheme 4): Hantzsch ester (**A**) undergoes a single-electron transfer process under the action of a photosensitizer, generating a cyclohexyl radical (**B**); subsequently, the cyclohexyl radical (**B**) undergoes an addition reaction with carbon monoxide in the system, further generating an intermediate acyl radical (**C**). Immediately afterward, the acyl radical (**C**) undergoes a radical coupling reaction by selectively capturing the aryl sulfur group in the thiosulfonate molecule, ultimately generating the target compound (**D**); meanwhile, the sulfonyl radical generated during the reaction can act as an oxidant to achieve the oxidative regeneration of the photosensitizer, thereby enabling the photosensitizer to continuously participate in the catalytic cycle and ensuring the efficient and stable progress of the reaction. The possibility of radical chain process can be excluded by the low yield (25%) that we obtained in the reaction with 10 h of irradiation and 14 h under dark.

**Scheme 4:**
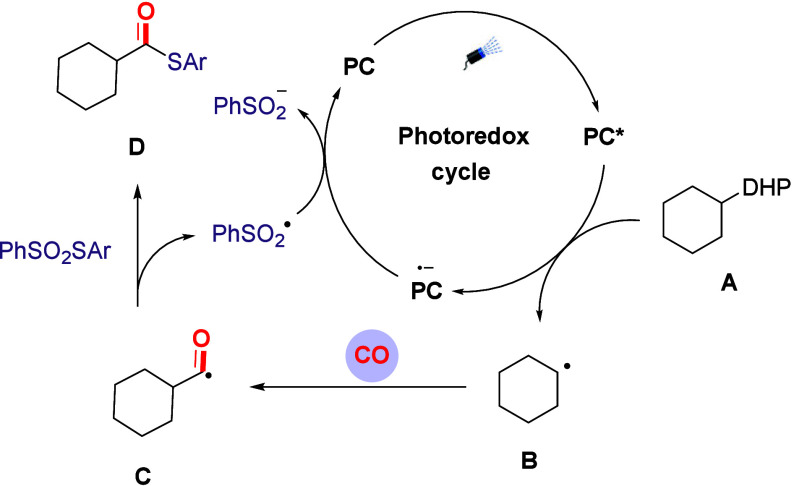
Proposed Mechanism

In summary, the present letter reports a novel visible-light-induced, metal-free-catalyzed method for the carbonylation synthesis of alkyl thioesters. This method utilizes Hantzsch ester compounds as alkyl radical precursors and, under a carbon monoxide atmosphere, selectively captures the aryl sulfur fragment from thiosulfonates through the formation of acyl radical intermediates, efficiently constructing alkyl thioester compounds. The reaction system functions under mild conditions, thus enabling the high-yield preparation of various thioester products. Furthermore, it exhibits excellent tolerance toward various functional groups under transition metal catalyst-free conditions. Additionally, thiosulfonates function as bifunctional reagents, serving both as sulfur sources to provide aryl sulfur fragments and as endogenous oxidants to facilitate the maintenance of the reaction cycle.

## Supplementary Material



## Data Availability

The data underlying this study are available in the published article and its Supporting Information.

## Notes

General comments, general procedure, optimization details, analytical data, and NMR spectra (PDF)
