# Supplementary material for: Visible-Light-Induced Carbonylative Synthesis of Aliphatic Thioesters
Source: Org Lett. 2026 Mar 26;28(14):4512–7. doi: 10.1021/acs.orglett.6c00753 (PMC13077695; doi:10.1021/acs.orglett.6c00753)

Supporting Information

Visible light-induced carbonylative synthesis of aliphatic thioesters

Ren-Guan Miao,† Ru-Han A† and Xiao-Feng Wu†*

† Dalian National Laboratory for Clean Energy, Dalian Institute of Chemical Physics, Chinese Academy of Sciences, 116023 Dalian, Liaoning, China, E-mail: xwu2020@dicp.ac.cn; Leibniz-Institut für Katalyse e.V., 18059 Rostock, Germany, E-mail: Xiao-Feng.Wu@catalysis.de.

Contents

1. General information 1
2. Optimization of the reaction conditions 2
3. General procedure for preparation of benzenesulfonothioates 4
4. General procedure for the synthesis of alkyl thioesters 4
5. Control experiments 6
6. General Procedure for scale-up reaction (1 mmol) 8
7. Synthetic applications 8
8. Characterization data of the corresponding product[1-2] 9
9. Reference 16
10. The NMR spectrum 17

1. General information

Unless otherwise noted, all reactions were carried out under N_2. All reagents were from commercial sources and used as received without further purification. All solvents were dried by standard techniques and distilled prior to use. Column chromatography was performed on silica gel (200-300 meshes) using petroleum ether (bp. 60~90 °C) as eluent. ^1H and ^{13}C NMR spectra were taken on 400 MHz or 700 MH)z instruments and spectral data were reported in ppm relative to tetramethylsilane (TMS) as the internal standard and $CDCl_3$ (^1H NMR δ 7.26, ^{13}C NMR δ 77.16) as solvent. All coupling constants (*J*) are reported in Hz with the following abbreviations: s = singlet, d = doublet, dd = double doublet, ddd = double doublet of doublets, t = triplet, dt = double triplet, q = quartet, m = multiplet, br = broad. All reactions were monitored by GC-FID or NMR analysis. HRMS data was obtained with Micromass HPLC-Q-TOF mass spectrometer (ESI-TOF) or Agilent 6540 Accurate-MS spectrometer (Q-TOF). Because of the high toxicity of carbon monoxide, all the reactions should be performed in an autoclave. The laboratory should be well-equipped with a CO detector and alarm system.

2. Optimization of the reaction conditions

Table S1. Optimization of solvents

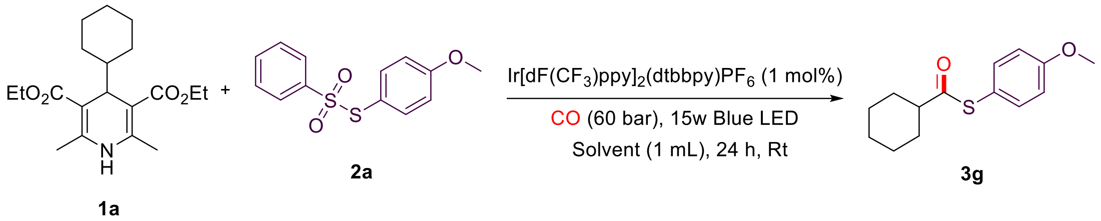

Entry	**Solvent**	Yield (%)
1	THF	40
2	DCM	20
3	DMAc	13
4	EA	35
5	MeCN	16
6	1,4-dioxane	26
7	DMF	17
8	Toluene	31
9	DME	35

Reaction conditions: **1a** (0.1 mmol), **2a** (0.15 mmol), Ir[dF(CF_3)ppy]$_2$(dtbbpy)PF_6 (1 mol%), Solvent (1 mL), CO (60 bar), Rt, 24 h, 15w Blue LEDs. Isolated yields.

Table S2. Optimization of substrate ratio

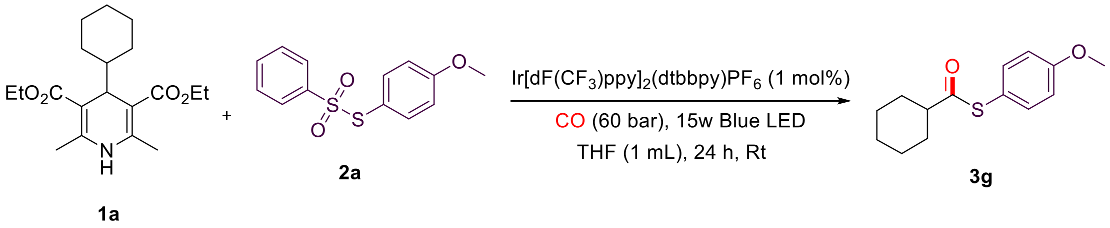

Entry	**1a : 2a (mmol)**	Yield (%)
1	0.15 : 0.1	56
2	0.2 : 0.1	58
3	0.25 : 0.1	61
4	0.3 : 0.1	59

Reaction conditions: **1a**, **2a**, Ir[dF(CF_3)ppy]$_2$(dtbbpy)PF_6 (1 mol%), THF (1 mL), CO

(60 bar), Rt, 24 h, 15w Blue LEDs. Isolated yields.

Table S3. Optimization of photocatalysts

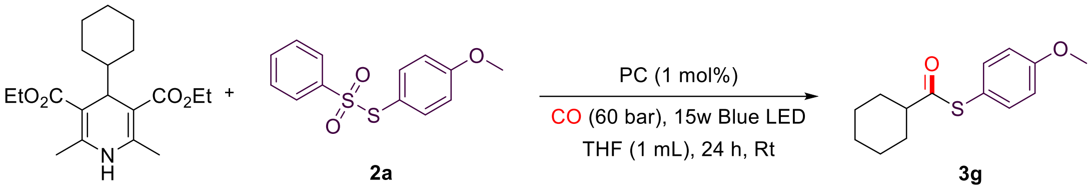

Entry	PC	Yield (%)
1	*fac*-(Irppy)$_3$	44
2	4-CzIPN	76
3	Ru(bpy)$_3$Cl$_2$·6H$_2$O	16
4[b]	Eosin Y	23
5	Ir(ppy)$_2$(dtbbpy)PF$_6$	61

Reaction conditions: **1a** (0.25 mmol), **2a** (0.1 mmol), photocatalysts (1 mol%), THF (1 mL), CO (60 bar), Rt, 24 h, 15w Blue LEDs. Isolated yields. [b]photocatalysts (5 mol%).

Table S4. Optimization of solvent dosage

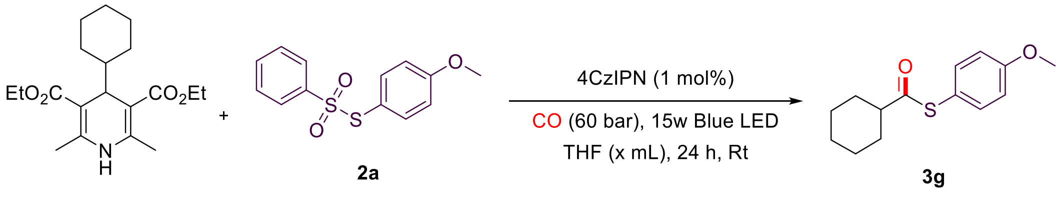

Entry	THF (x mL)	Yield (%)
1	0.5	25
2	1.5	56
3	2	80
4	2.5	85
5[b]	2.5	70

Reaction conditions: **1a** (0.25 mmol), **2a** (0.1 mmol), 4CzIPN (1 mol%), THF (x mL), CO (60 bar), Rt, 24 h, 15w Blue LEDs. Isolated yields. [b] CO (40 bar).

3. General procedure for preparation of benzenesulfonothioates

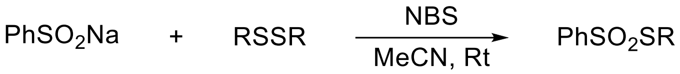

A mixture of sodium sulfinate (4.0 mmol), disulfide (1.0 mmol) and *N*-bromosuccinimide (2.0 mmol) in acetonitrile (15.0 mL) was stirred at room temperature for 15 h. Subsequently, the solvent was evaporated, the residue was redisolved in EtOAc, washed with water and extracted with EtOAc (3x15 mL). The combined organic phases were dried over $MgSO_4$. After filtration and concentration, the residue was purified by flash chromatography.

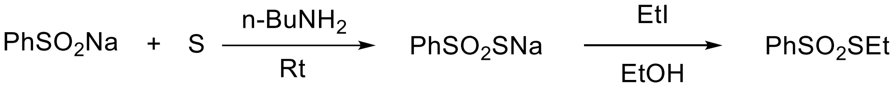

A mixture of $PhSO_2Na$ (1.0 equiv.) and S (1.0 equiv.) in $^{n}BuNH_2$ (1.0 mmol/mL) was stirred atroom temperature for 0.5 h. After removal of the solvent under reduced pressure, the residue was washed by Et_2O to obtain a white solid $PhSO_2SNa$. Then $PhSO_2SNa$ was dissolved in EtOH (1.0 mmol/mL), then C_2H_5I (2.0 equiv.) was added to the solution. The reaction mixture was stirred at 40-45 ^{o}C for 24 h. After removal of the solvent under reduced pressure, the reaction mixture was poured on a solution of $Na_2S_2O_3$ and CH_2Cl_2. The precipitate was filtered and dried by anhydrous Na_2SO_4, the residue was purified through column chromatography afforded the desired product as a yellow oil.

4. General procedure for the synthesis of alkyl thioesters

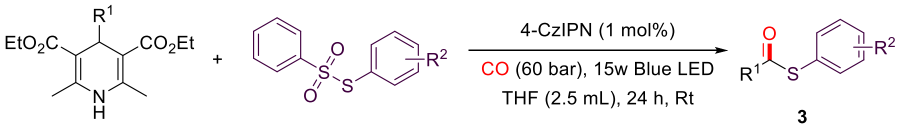

1 2 3

A 4 mL screw-cap vial was charged with **1** (0.25 mmol), **2** (0.1 mmol), 4-CzIPN (1 mol%) and an oven-dried stirring bar. The vial was closed with a Teflon septum and cap and connected to the atmosphere via a needle. Then THF (2.5 mL) was added with a syringe under N_2 atmosphere. The closed autoclave was flushed two times with

nitrogen (~ 10 bar), and a pressure of 60 bar CO was charged at room temperature. The reaction mixture was stirred at room temperature under 15w Blue LEDs (450-460 nm) for 24 hours. After the reaction, the pressure was released carefully. The mixture was concentrated under vacuum. The crude product was purified by column chromatography (PE/EA = 50/1) on silica gel to afford the corresponding products.

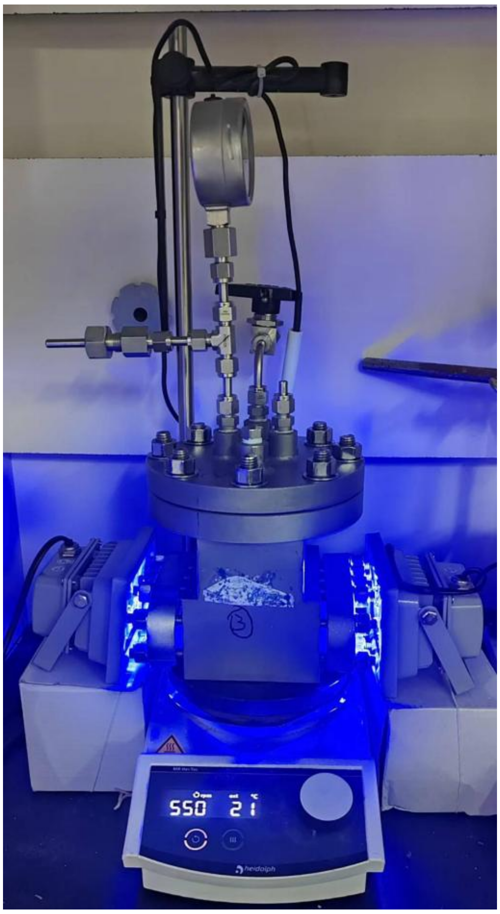

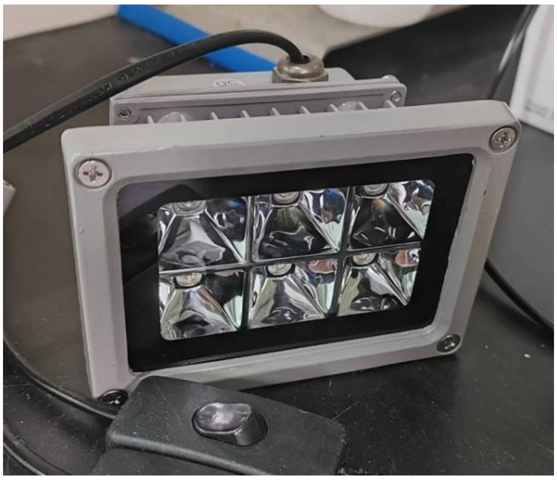

China Shenzhen boxing Tech. (BS-GS-UV-450-460NM-00), 100-260V, LED, 15W, 450-460 nm

5. Control experiments

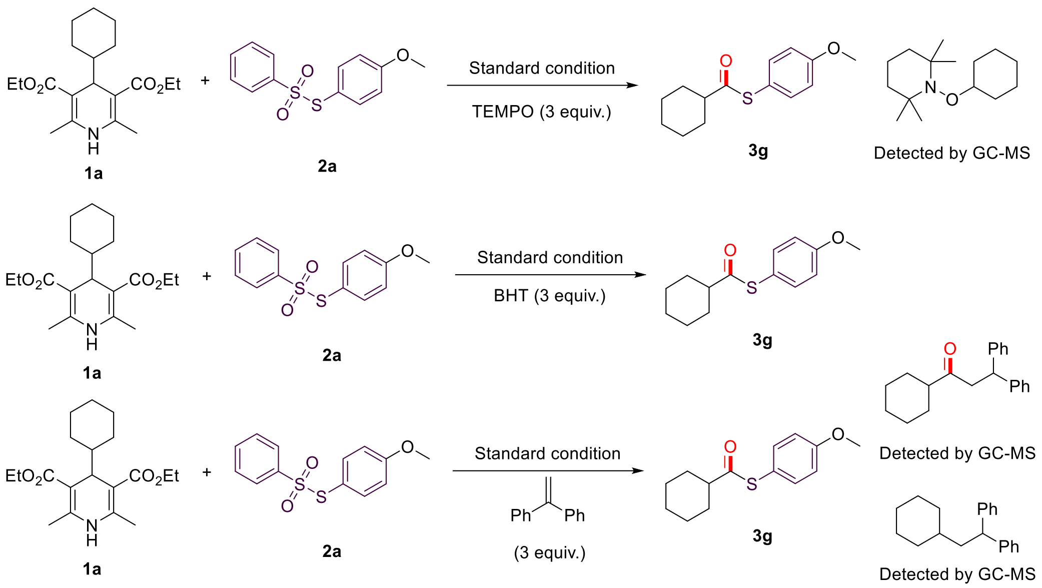

A 4 mL screw-cap vial was charged with **1a** (0.2 mmol), **2g** (0.1 mmol), 4CzIPN (1 mol%), radical scavenger (3 equiv.) and an oven-dried stirring bar. The vial was closed with a Teflon septum and cap and connected to the atmosphere via a needle. Then THF (2.5 mL) was added with a syringe under N_2 atmosphere. The closed autoclave was flushed two times with nitrogen (~ 10 bar), and a pressure of 60 bar CO was charged at room temperature. The reaction mixture was stirred at room temperature under 15w Blue LEDs (450-460 nm) for 24 hours.

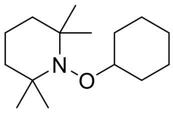

1-(cyclohexyloxy)-2,2,6,6-tetramethylpiperidine

HRMS (ESI-TOF): calcd for $[M+H]^+$ $C_{15}H_{30}NO^+$ 240.2322; Found: 240.2323.

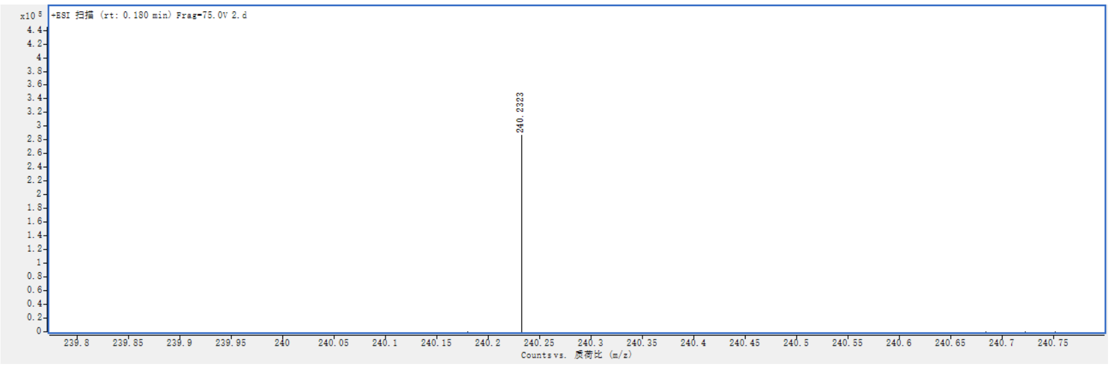

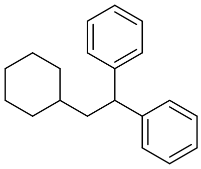

(2-cyclohexylethane-1,1-diyl)dibenzene

HRMS (ESI-TOF): calcd for $[M+H]^+$ $C_{20}H_{25}^+$ 265.1951; Found: 265.1941.

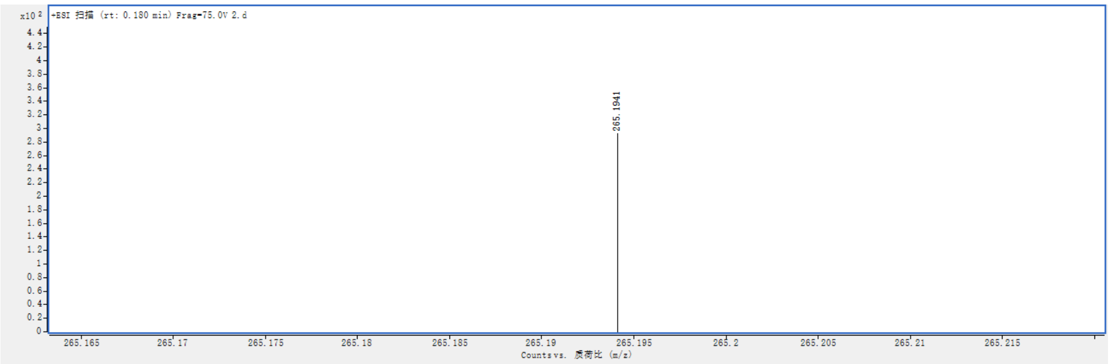

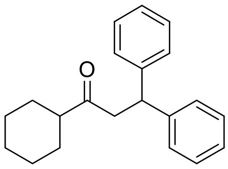

1-cyclohexyl-3,3-diphenylpropan-1-one

HRMS (ESI-TOF): calcd for $[M+H]^+$ $C_{21}H_{25}O^+$ 293.1900; Found: 293.1894.

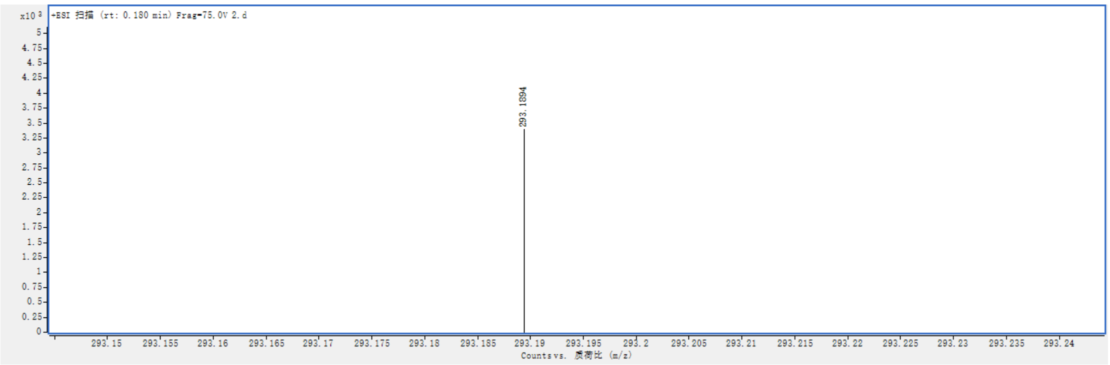

6. General Procedure for scale-up reaction (1 mmol scale)

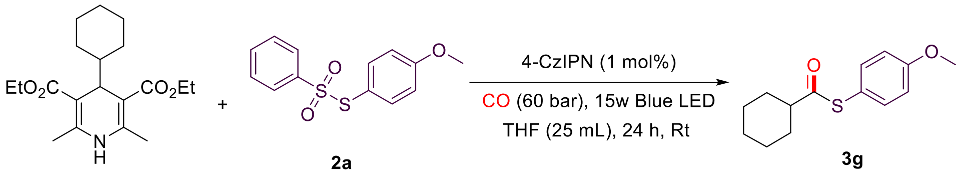

1a 2a 3g

A 40 mL screw-cap vial was charged with **1a** (2.5 mmol, 837.5 mg, 2.5 equiv.), **2a** (1 mmol, 280 mg, 1 equiv.), 4CzIPN (1 mol%, 7.9 mg) and an oven-dried stirring bar. The vial was closed with a Teflon septum and cap and connected to the atmosphere via a needle. Then THF (25 mL) was added with a syringe under N_2 atmosphere. The closed autoclave was flushed two times with nitrogen (~ 10 bar), and a pressure of 60 bar CO were charged. The reaction mixture was stirred at room temperature under 15w Blue LEDs (450-460 nm) for 24 hours. After the reaction, the pressure was released carefully. The mixture was concentrated under vacuum. The crude product was purified by column chromatography (PE/EA = 50/1) on silica gel to afford the corresponding product **3g** in 70% yield (175 mg).

7. Synthetic applications

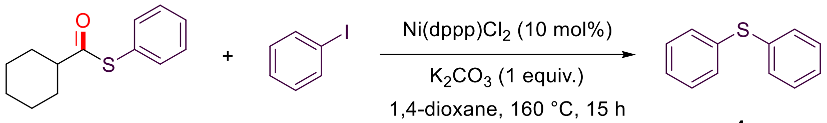

3a 4a

An oven-dried pressure vial equipped with a stir bar was charged with **3a** (44 mg, 0.20 mmol, 1.0 equiv), Ni(dppp)Cl_2 (10 mol%) and K_2CO_3 (41.5 mg, 1.5 equiv), placed under a positive pressure of N_2 and subjected to three evacuation/backfilling cycles under high vacuum. Iodobenzene (57.1 mg, 0.28mmol), 1,4-dioxane (1.0 mL) were added with vigorous stirring at room temperature, the vial was tightly capped and was placed in a preheated oil bath at 160 °C, stirred for 15 h at 160 °C. The crude residue was purified by flash column chromatography to afford the title compound as a colorless liquid.

8. Characterization data of the corresponding product

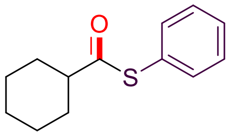

***S*-phenyl cyclohexanecarbothioate**

The reaction solution was processed as general experimental procedure to afford the corresponding product **3a** as a colorless liquid (19.6 mg, 89%).[1]

^{1}H NMR (400 MHz, $CDCl_3$) δ 7.40 (s, 5H), 2.70 – 2.49 (m, 1H), 2.00 (d, J = 12.6 Hz, 2H), 1.88 – 1.74 (m, 2H), 1.68 (d, J = 11.0 Hz, 1H), 1.56 – 1.42 (m, 2H), 1.35 – 1.17 (m, 3H).

^{13}C NMR (101 MHz, $CDCl_3$) δ 200.8, 134.6, 129.2, 129.1, 128.0, 52.5, 29.6, 25.6, 25.5.

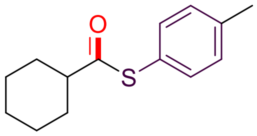

***S*-(*p*-tolyl) cyclohexanecarbothioate**

The reaction solution was processed as general experimental procedure to afford the corresponding product **3b** as a colorless liquid (19.0 mg, 81%).[2]

^{1}H NMR (400 MHz, $CDCl_3$) δ 7.33 (t, J = 22.8 Hz, 1H), 7.27 (d, J = 1.8 Hz, 1H), 7.21 (d, J = 8.0 Hz, 2H), 2.60 (tt, J = 11.4, 3.5 Hz, 1H), 2.37 (s, 3H), 2.00 (dd, J = 13.2, 2.7 Hz, 2H), 1.89 – 1.77 (m, 2H), 1.72 – 1.64 (m, 1H), 1.54 (dt, J = 11.9, 4.4 Hz, 2H), 1.38 – 1.18 (m, 3H).

^{13}C NMR (101 MHz, $CDCl_3$) δ 201.4, 139.5, 134.7, 130.1, 124.5, 52.6, 29.7, 25.7, 25.6, 21.4.

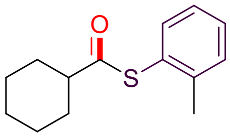

***S*-(*o*-tolyl) cyclohexanecarbothioate**

The reaction solution was processed as general experimental procedure to afford the corresponding product **3c** as a colorless liquid (22.0 mg, 94%).[3]

^{1}H NMR (400 MHz, CDCl$_3$) δ 7.41 – 7.36 (m, 1H), 7.35 – 7.29 (m, 2H), 7.24 – 7.17 (m, 1H), 2.71 – 2.53 (m, 1H), 2.33 (s, 3H), 2.07 – 1.96 (m, 2H), 1.90 – 1.78 (m, 2H), 1.75 – 1.64 (m, 1H), 1.62 – 1.49 (m, 2H), 1.41 – 1.19 (m, 3H).

^{13}C NMR (101 MHz, CDCl$_3$) δ 200.5, 142.1, 136.2, 130.8, 130.0, 127.5, 126.6, 52.7, 29.7, 25.7, 25.6, 20.8.

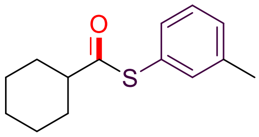

***S*-(*m*-tolyl) cyclohexanecarbothioate**

The reaction solution was processed as general experimental procedure to afford the corresponding product **3d** as a colorless liquid (18.7 mg, 80%).

^{1}H NMR (400 MHz, CDCl$_3$) δ 7.29 (t, J = 7.5 Hz, 1H), 7.21 (dd, J = 7.9, 6.0 Hz, 3H), 2.72 – 2.51 (m, 1H), 2.36 (s, 3H), 2.01 (dd, J = 12.3, 3.5 Hz, 2H), 1.87 – 1.77 (m, 2H), 1.73 – 1.64 (m, 1H), 1.57 – 1.46 (m, 2H), 1.40 – 1.19 (m, 3H).

^{13}C NMR (101 MHz, CDCl$_3$) δ 201.2, 139.1, 135.3, 131.8, 130.2, 129.0, 127.7, 52.6, 29.7, 25.7, 25.6, 21.4.

HRMS (ESI-TOF): calcd for $[M+H]^+$ $C_{14}H_{19}OS^+$ 235.1151; Found: 235.1160.

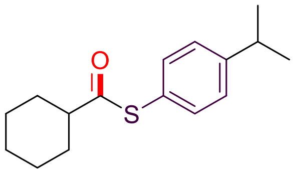

***S*-(4-isopropylphenyl) cyclohexanecarbothioate**

The reaction solution was processed as general experimental procedure to afford the corresponding product **3e** as a colorless liquid (19.1 mg, 73%).

^{1}H NMR (400 MHz, CDCl$_3$) δ 7.36 (d, J = 28.8 Hz, 1H), 7.30 (s, 1H), 7.28 – 7.23 (m, 2H), 3.03 – 2.77 (m, 1H), 2.71 – 2.50 (m, 1H), 1.99 (d, J = 10.8 Hz, 2H), 1.87 – 1.75 (m, 2H), 1.67 (d, J = 10.8 Hz, 1H), 1.55 – 1.45 (m, 2H), 1.36 – 1.15 (m, 9H).

^{13}C NMR (101 MHz, CDCl$_3$) δ 201.4, 150.2, 134.7, 127.5, 124.8, 52.6, 34.1, 29.7, 25.7, 25.6, 24.0.

HRMS (ESI-TOF): calcd for $[M+H]^+$ $C_{16}H_{23}OS^+$ 263.1464; Found: 263.1468.

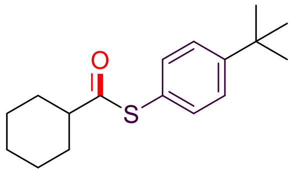

_S_-(4-(_tert_-butyl)phenyl) cyclohexanecarbothioate

The reaction solution was processed as general experimental procedure to afford the corresponding product **3f** as a colorless liquid (19.3 mg, 70%).[1]

^{1}H NMR (400 MHz, $CDCl_3$) δ 7.42 (t, J = 6.7 Hz, 2H), 7.33 (d, J = 8.3 Hz, 2H), 2.65 – 2.56 (m, 1H), 2.08 – 1.96 (m, 2H), 1.88 – 1.78 (m, 2H), 1.73 – 1.63 (m, 1H), 1.61 – 1.46 (m, 2H), 1.40 – 1.23 (m, 12H).

^{13}C NMR (101 MHz, $CDCl_3$) δ 201.4, 152.5, 134.4, 126.4, 124.6, 52.6, 34.9, 31.4, 29.7, 25.8, 25.6.

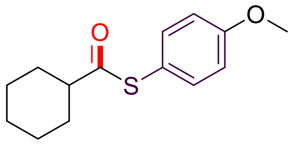

_S_-(4-methoxyphenyl) cyclohexanecarbothioate

The reaction solution was processed as general experimental procedure to afford the corresponding product **3g** as a colorless liquid (21.2 mg, 85%).[3]

^{1}H NMR (400 MHz, $CDCl_3$) δ 7.43 – 7.27 (m, 2H), 6.98 – 6.78 (m, 2H), 3.82 (s, 3H), 2.70 – 2.45 (m, 1H), 1.99 (dd, J = 13.2, 2.5 Hz, 2H), 1.90 – 1.78 (m, 2H), 1.72 – 1.62 (m, 1H), 1.60 – 1.44 (m, 2H), 1.39 – 1.22 (m, 3H).

^{13}C NMR (101 MHz, $CDCl_3$) δ 201.9, 160.6, 136.3, 118.8, 114.9, 55.5, 52.4, 29.7, 25.8, 25.6.

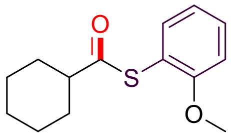

_S_-(2-methoxyphenyl) cyclohexanecarbothioate

The reaction solution was processed as general experimental procedure to afford the corresponding product **3h** as a colorless liquid (24.0 mg, 96%).

^{1}H NMR (400 MHz, CDCl$_3$) δ 7.46 – 7.34 (m, 2H), 7.04 – 6.90 (m, 2H), 3.84 (s, 3H), 2.75 – 2.52 (m, 1H), 2.02 (dd, J = 13.2, 2.7 Hz, 2H), 1.85 – 1.74 (m, 2H), 1.76 – 1.63 (m, 1H), 1.60 – 1.46 (m, 2H), 1.41 – 1.23 (m, 3H).

^{13}C NMR (101 MHz, CDCl$_3$) δ 200.2, 159.4, 137.0, 131.5, 121.2, 116.4, 111.6, 56.1, 52.6, 29.7, 25.8, 25.6.

HRMS (ESI-TOF): calcd for $[M+H]^+$ $C_{14}H_{19}O_2S^+$ 251.1100; Found: 251.1108.

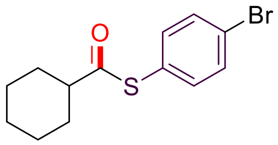

***S*-(4-bromophenyl) cyclohexanecarbothioate**

The reaction solution was processed as general experimental procedure to afford the corresponding product **3i** as a colorless liquid (28.0 mg, 94%).[2]

^{1}H NMR (400 MHz, CDCl$_3$) δ 7.56 – 7.47 (m, 2H), 7.29 – 7.20 (m, 2H), 2.67 – 2.52 (m, 1H), 1.99 (dd, J = 13.1, 2.8 Hz, 2H), 1.90 – 1.75 (m, 2H), 1.74 – 1.62 (m, 1H), 1.60 – 1.41 (m, 2H), 1.41 – 1.17 (m, 3H).

^{13}C NMR (101 MHz, CDCl$_3$) δ 200.2, 136.2, 132.4, 127.2, 123.9, 52.7, 29.6, 25.7, 25.6.

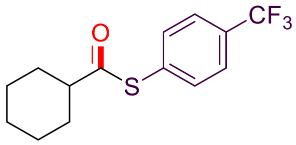

***S*-(4-(trifluoromethyl)phenyl) cyclohexanecarbothioate**

The reaction solution was processed as general experimental procedure to afford the corresponding product **3j** as a colorless liquid (14.4 mg, 50%).[2]

^{1}H NMR (400 MHz, CDCl$_3$) δ 7.64 (d, J = 8.2 Hz, 2H), 7.52 (d, J = 8.0 Hz, 2H), 2.75 – 2.48 (m, 1H), 2.01 (dd, J = 13.1, 2.8 Hz, 2H), 1.90 – 1.78 (m, 2H), 1.69 (dd, J = 11.7, 4.0 Hz, 1H), 1.56 – 1.51 (m, 2H), 1.36 – 1.23 (m, 3H).

^{13}C NMR (176 MHz, CDCl$_3$) δ 199.5, 134.7, 131.1 (q, J_{C-F} = 32.7 Hz), 129.1, 125.8 (q, J_{C-F} = 3.7 Hz), 123.9 (q, J_{C-F} = 272.2 Hz), 52.8, 29.5, 25.5, 25.4.

^{19}F NMR (376 MHz, CDCl$_3$) δ -62.8.

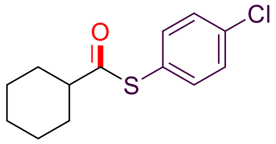

***S*-(4-chlorophenyl) cyclohexanecarbothioate**

The reaction solution was processed as general experimental procedure to afford the corresponding product **3k** as a colorless liquid (15.2 mg, 60%).[2]

^{1}H NMR (400 MHz, $CDCl_3$) δ 7.41 – 7.35 (m, 2H), 7.35 – 7.28 (m, 2H), 2.68 – 2.47 (m, 1H), 2.00 (dd, J = 13.2, 2.5 Hz, 2H), 1.86 – 1.77 (m, 2H), 1.68 (dd, J = 11.1, 3.5 Hz, 1H), 1.58 – 1.45 (m, 2H), 1.36 – 1.22 (m, 3H).

^{13}C NMR (101 MHz, $CDCl_3$) δ 200.4, 136.0, 135.7, 129.5, 126.6, 52.7, 29.6, 25.7, 25.6.

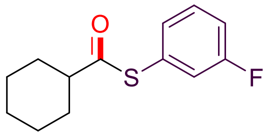

***S*-(3-fluorophenyl) cyclohexanecarbothioate**

The reaction solution was processed as general experimental procedure to afford the corresponding product **3l** as a colorless liquid (14.8 mg, 62%).

^{1}H NMR (400 MHz, $CDCl_3$) δ 7.40 – 7.33 (m, 1H), 7.20 – 7.06 (m, 3H), 2.67 – 2.52 (m, 1H), 2.00 (dd, J = 13.2, 2.6 Hz, 2H), 1.88 – 1.76 (m, 2H), 1.73 – 1.63 (m, 1H), 1.58 – 1.46 (m, 2H), 1.38 – 1.22 (m, 3H).

^{13}C NMR (101 MHz, $CDCl_3$) δ 200.1, 162.7 (d, J_{C-F} = 248.9 Hz), 130.4 (d, J_{C-F} = 8.5 Hz), 130.3 (d, J_{C-F} = 3.4 Hz), 130.0 (d, J_{C-F} = 8.1 Hz), 121.6 (d, J_{C-F} = 22.5 Hz), 116.4 (d, J_{C-F} = 21.0 Hz), 52.8, 29.6, 25.7, 25.6.

^{19}F NMR (376 MHz, $CDCl_3$) δ -111.9.

HRMS (ESI-TOF): calcd for $[M+H]^+$ $C_{13}H_{16}FOS^+$ 239.0900; Found: 239.0906.

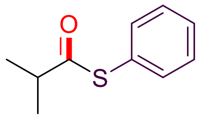

***S*-phenyl 2-methylpropanethioate**

The reaction solution was processed as general experimental procedure to afford the corresponding product **3p** as a colorless liquid (11.7 mg, 65%).[1]

^{1}H NMR (400 MHz, CDCl$_3$) δ 7.41 (s, 5H), 1.27 (d, J = 6.9 Hz, 7H).

^{13}C NMR (101 MHz, CDCl$_3$) δ 202.0, 134.7, 129.3, 129.2, 128.0, 43.1, 19.5.

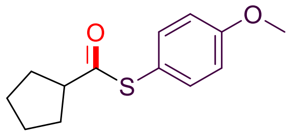

***S*-(4-methoxyphenyl) cyclopentanecarbothioate**

The reaction solution was processed as general experimental procedure to afford the corresponding product **3q** as a colorless liquid (19.6 mg, 83%).

^{1}H NMR (400 MHz, CDCl$_3$) δ 7.31 (d, J = 8.5 Hz, 2H), 6.93 (d, J = 8.5 Hz, 2H), 3.82 (s, 3H), 3.17 – 3.01 (m, 1H), 2.01 – 1.83 (m, 4H), 1.79 – 1.67 (m, 2H), 1.66 – 1.57 (m, 2H).

^{13}C NMR (101 MHz, CDCl$_3$) δ 202.1, 160.6, 136.3, 119.0, 114.9, 55.5, 52.8, 30.8, 26.1.

HRMS (ESI-TOF): calcd for $[M+H]^+$ $C_{14}H_{17}O_2S^+$ 237.0944; Found: 237.0949.

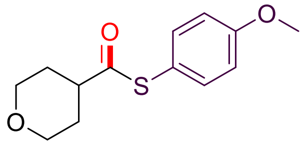

***S*-(4-methoxyphenyl) tetrahydro-2H-pyran-4-carbothioate**

The reaction solution was processed as general experimental procedure to afford the corresponding product **3r** as a colorless liquid (20.2 mg, 80%).

^{1}H NMR (700 MHz, CDCl$_3$) δ 7.30 (d, J = 8.6 Hz, 2H), 6.94 (d, J = 8.6 Hz, 2H), 4.02 (d, J = 11.6 Hz, 2H), 3.82 (s, 3H), 3.55 – 3.35 (m, 2H), 2.89 – 2.78 (m, 1H), 1.91 – 1.82 (m, 4H).

^{13}C NMR (176 MHz, CDCl$_3$) δ 200.4, 160.8, 136.3, 118.0, 115.0, 67.2, 55.5, 49.0, 29.3.

HRMS (ESI-TOF): calcd for $[M+H]^+$ $C_{13}H_{17}O_3S^+$ 253.0893; Found: 253.0899.

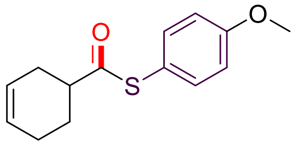

***S*-(4-methoxyphenyl) cyclohex-3-ene-1-carbothioate**

The reaction solution was processed as general experimental procedure to afford the

corresponding product **3s** as a colorless liquid (19.8 mg, 80%).

^{1}H NMR (400 MHz, CDCl$_3$) δ 7.32 (d, J = 8.6 Hz, 2H), 6.94 (d, J = 8.6 Hz, 2H), 5.71 (s, 2H), 3.82 (s, 3H), 2.96 – 2.62 (m, 1H), 2.37 – 2.26 (m, 2H), 2.21 – 2.01 (m, 3H), 1.83 – 1.68 (m, 1H).

^{13}C NMR (101 MHz, CDCl$_3$) δ 201.7, 160.7, 136.3, 126.8, 125.1, 118.5, 115.0, 55.5, 48.4, 28.1, 25.9, 24.7.

HRMS (ESI-TOF): calcd for $[M+H]^+$ $C_{14}H_{17}O_2S^+$ 249.0944; Found: 249.0948.

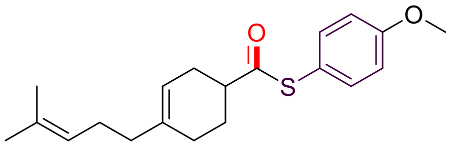

***S*-(4-methoxyphenyl) 4-(4-methylpent-3-en-1-yl)cyclohex-3-ene-1-carbothioate**

The reaction solution was processed as general experimental procedure to afford the corresponding product **3t** as a colorless liquid (19.8 mg, 60%).

^{1}H NMR (400 MHz, CDCl$_3$) δ 7.31 (d, J = 8.8 Hz, 2H), 6.93 (d, J = 8.8 Hz, 2H), 5.41 (s, 1H), 5.09 (t, J = 6.8 Hz, 1H), 3.82 (s, 3H), 2.79 (dd, J = 17.5, 7.9 Hz, 1H), 2.38 – 2.24 (m, 2H), 2.14 – 1.88 (m, 8H), 1.69 (s, 3H), 1.61 (s, 3H).

^{13}C NMR (101 MHz, CDCl$_3$) δ 201.9, 160.7, 137.7, 136.3, 131.7, 124.3, 118.8, 118.6, 115.0, 55.5, 48.6, 37.6, 28.4, 28.0, 26.5, 26.3, 25.8, 17.8.

HRMS (ESI-TOF): calcd for $[M+H]^+$ $C_{20}H_{27}O_2S^+$ 331.1726; Found: 331.1732.

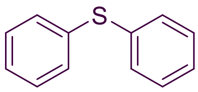

diphenylsulfane

The reaction solution was processed as general experimental procedure to afford the corresponding product **4a** as a colorless liquid (29.8 mg, 80%).[2]

^{1}H NMR (400 MHz, CDCl$_3$) δ 7.36 – 7.29 (m, 4H), 7.26 (t, J = 7.3 Hz, 4H), 7.20 (dd, J = 8.3, 6.0 Hz, 2H).

^{13}C NMR (101 MHz, CDCl$_3$) δ 135.9, 131.1, 129.3, 127.1.

9. Reference

[1] Chen, B.; Wu, X.-F. Manganese(III)-promoted thiocarbonylation of alkylborates with disulfides: synthesis of aliphatic thioesters. *Org. Biomol. Chem.* **2021**, *19*, 9654-9658.

[2] Wang, L.-C.; Yang, H.; Li, Q.; Wu, X.-F. Streamlined Carbonylation of Csp^3–H Bonds: Divergent Synthesis of Diverse Carbonyl Compounds. Streamlined Carbonylation of Csp^3–H Bonds: Divergent Synthesis of Diverse Carbonyl Compounds. *ACS Catal.* **2025**, *15*, 10039-10050.

[3] Cao, H.; Shi, Y.; Ma, J.; Yan, P.; Cong, X.; Bie, F. Palladium- and nickel-catalyzed synthesis of thioethers via thioesters – Aryl halides coupling. *Tetrahedron Lett.*, **2023**, *119*, 154414.

10. The NMR spectrum

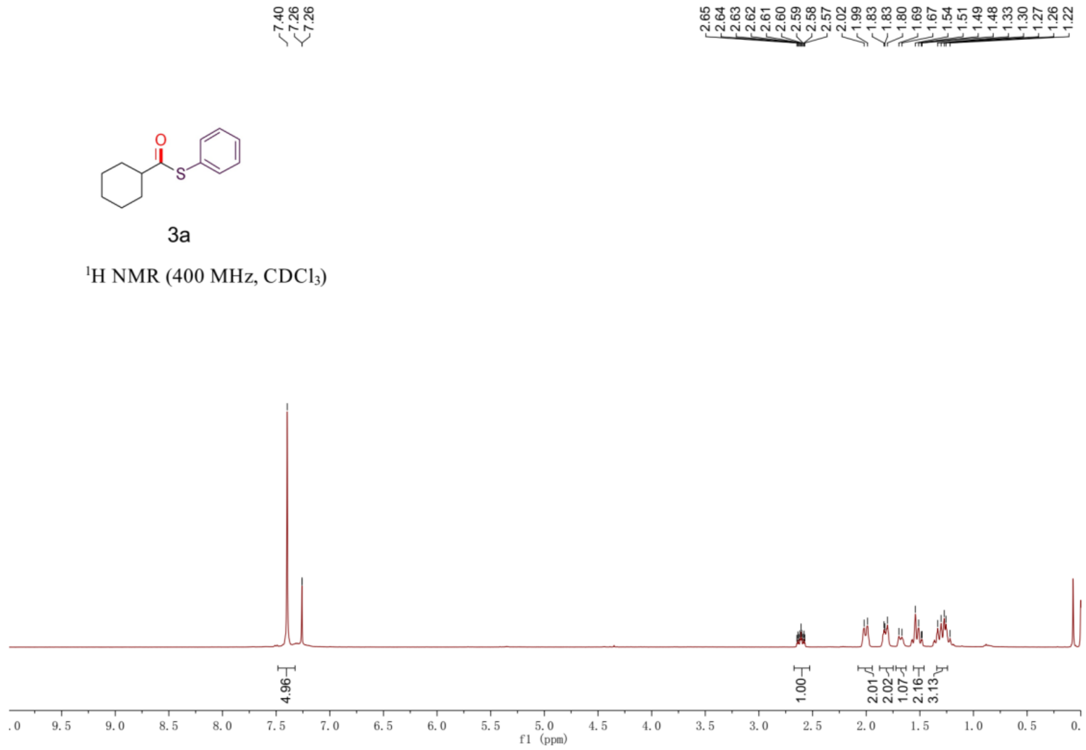

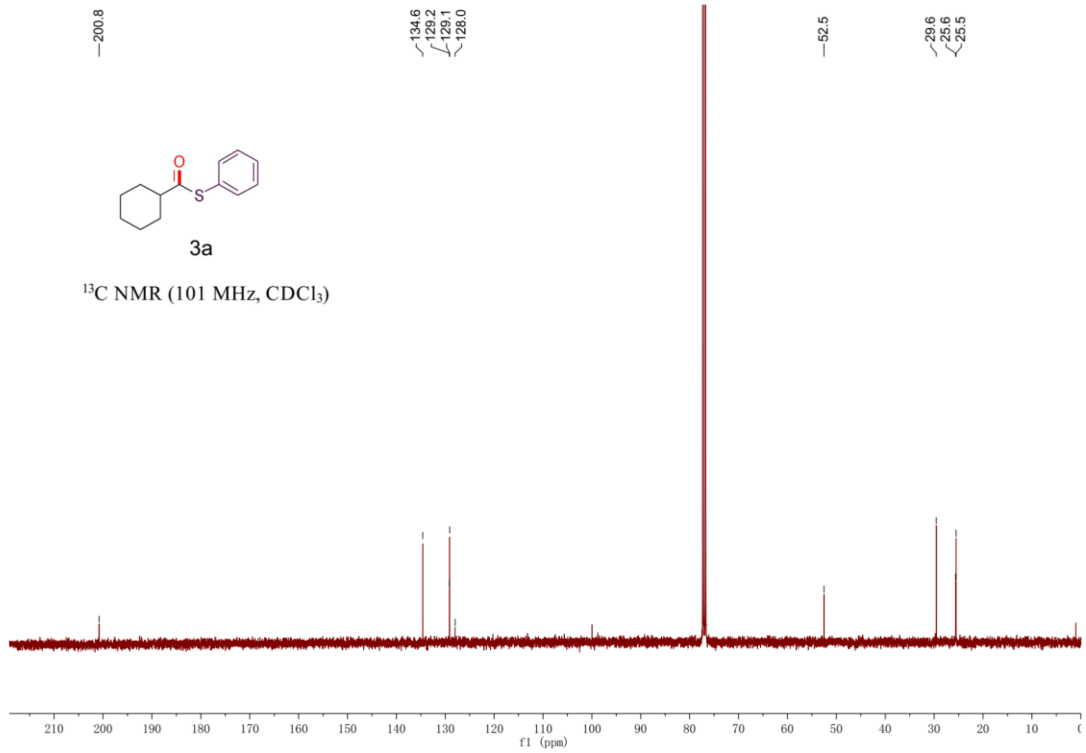

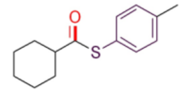

3b

^{1}H NMR (400 MHz, $CDCl_3$)

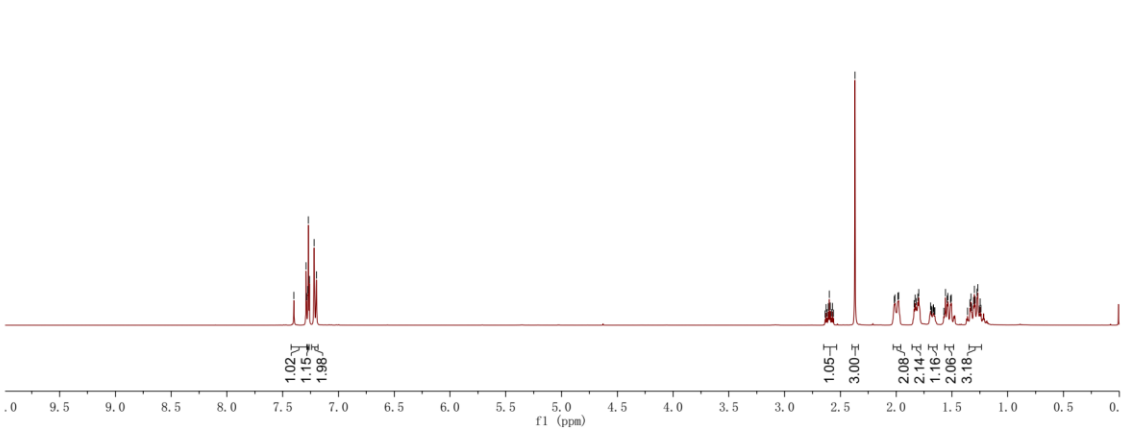

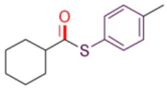

3b

^{13}C NMR (101 MHz, $CDCl_3$)

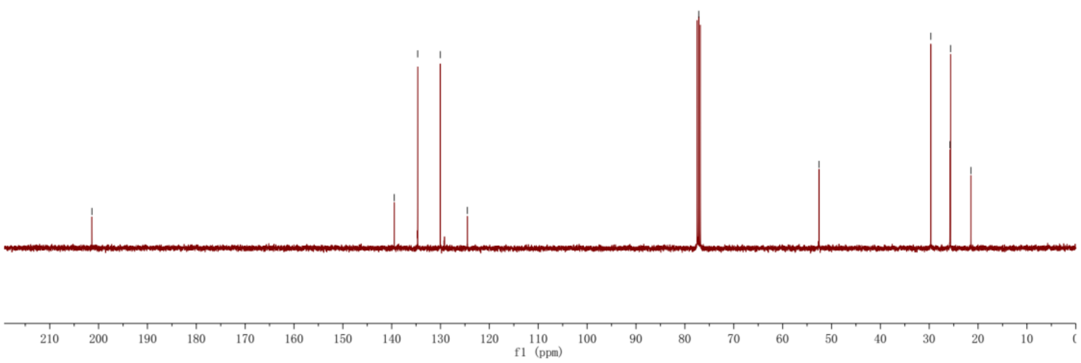

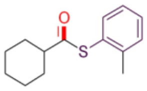

3c

^{1}H NMR (400 MHz, $CDCl_3$)

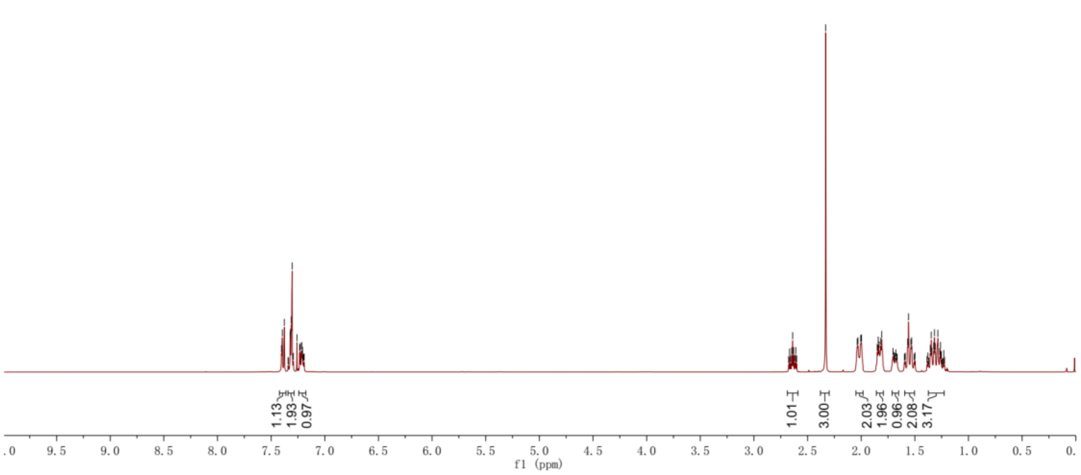

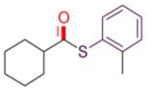

3c

^{13}C NMR (101 MHz, $CDCl_3$)

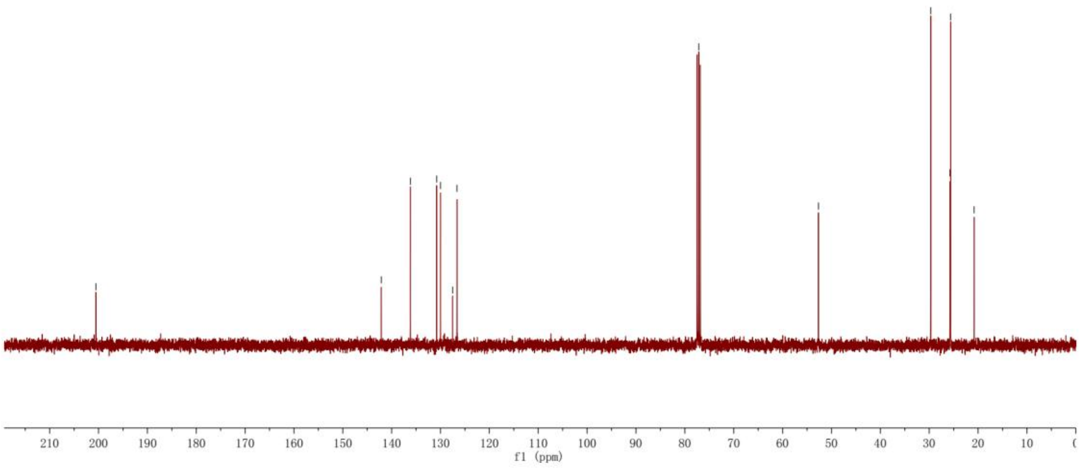

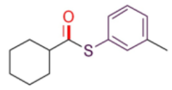

3d

^{1}H NMR (400 MHz, CDCl3)

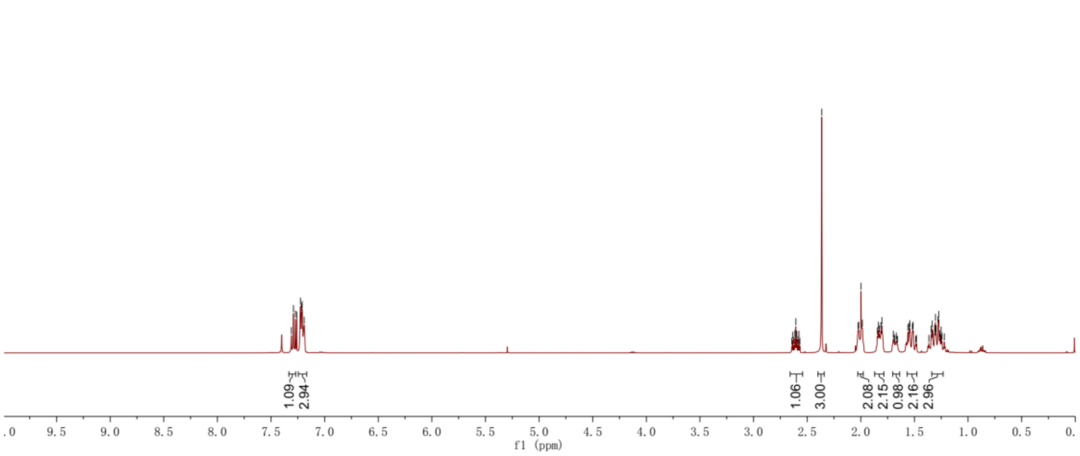

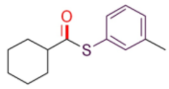

3d

^{13}C NMR (101 MHz, CDCl3)

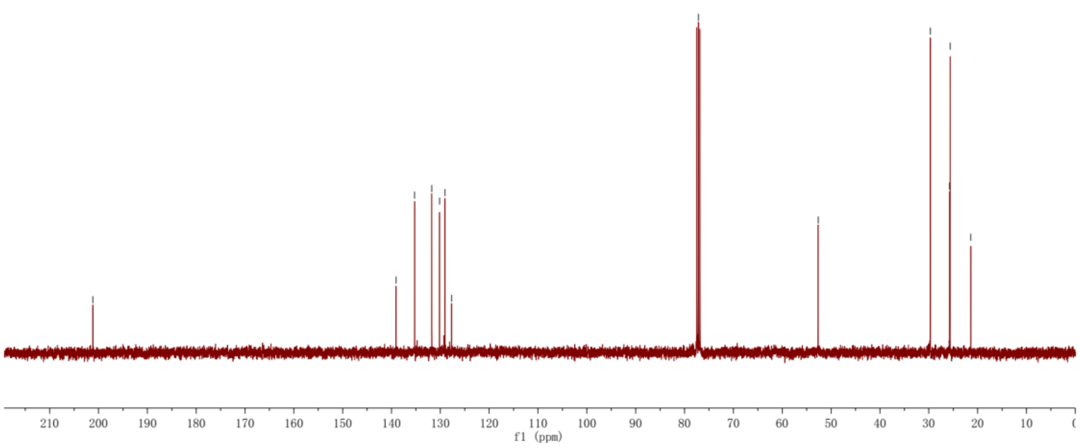

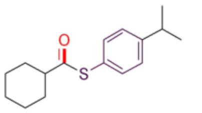

3e

^{1}H NMR (400 MHz, CDCl$_3$)

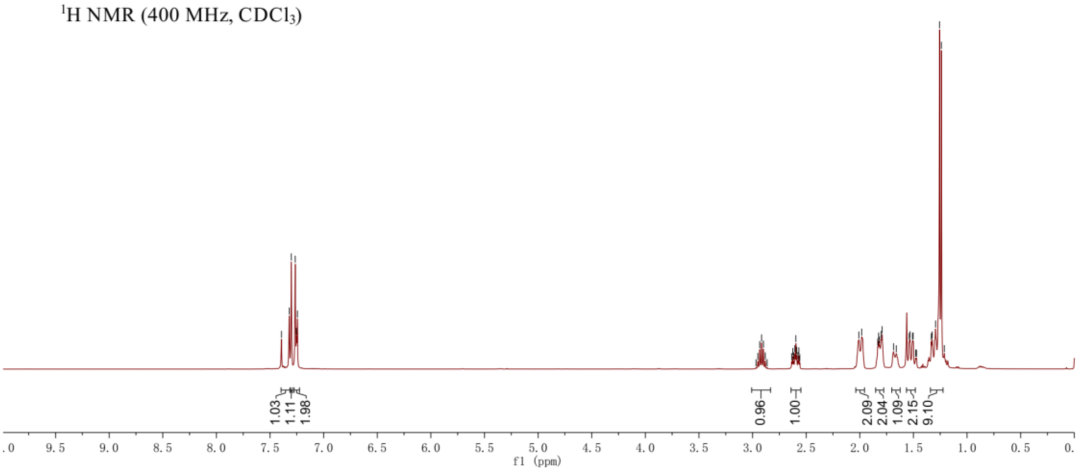

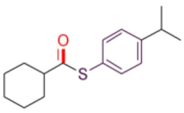

3e

^{13}C NMR (101 MHz, CDCl$_3$)

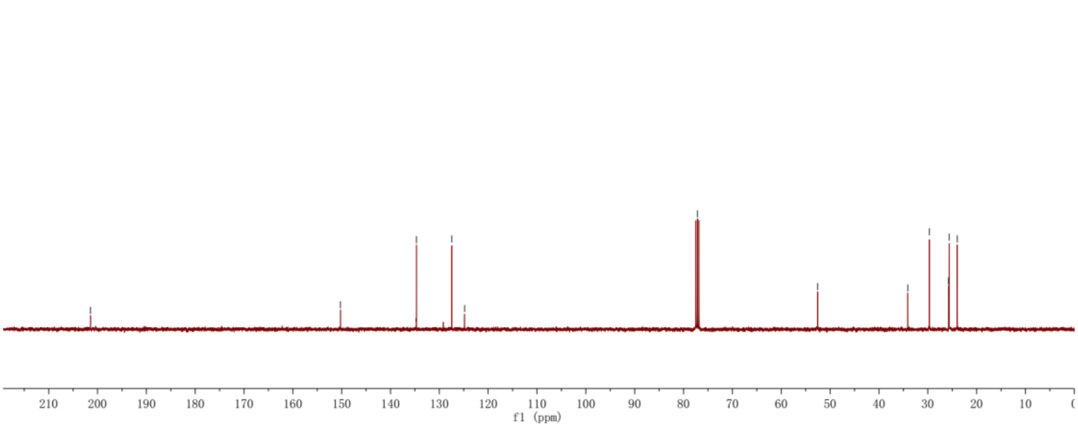

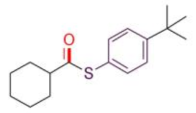

3f

^{1}H NMR (400 MHz, $CDCl_3$)

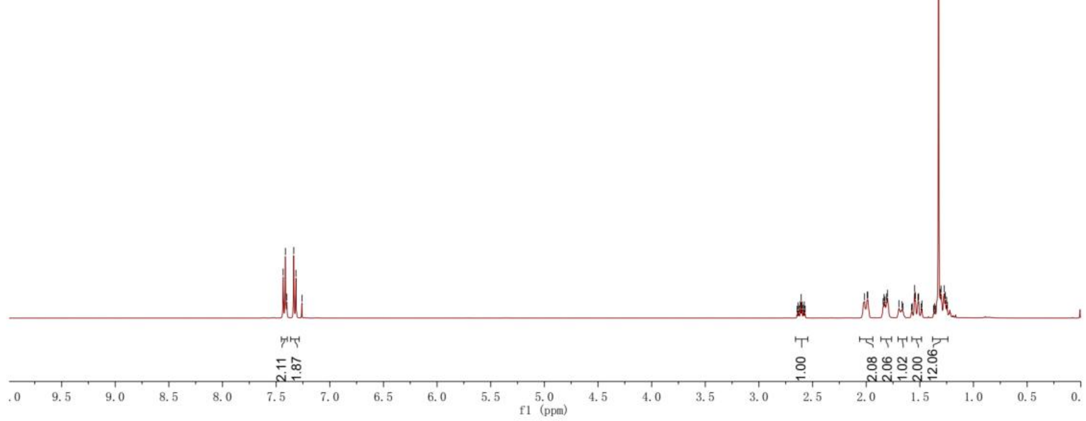

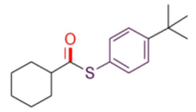

3f

^{13}C NMR (101 MHz, $CDCl_3$)

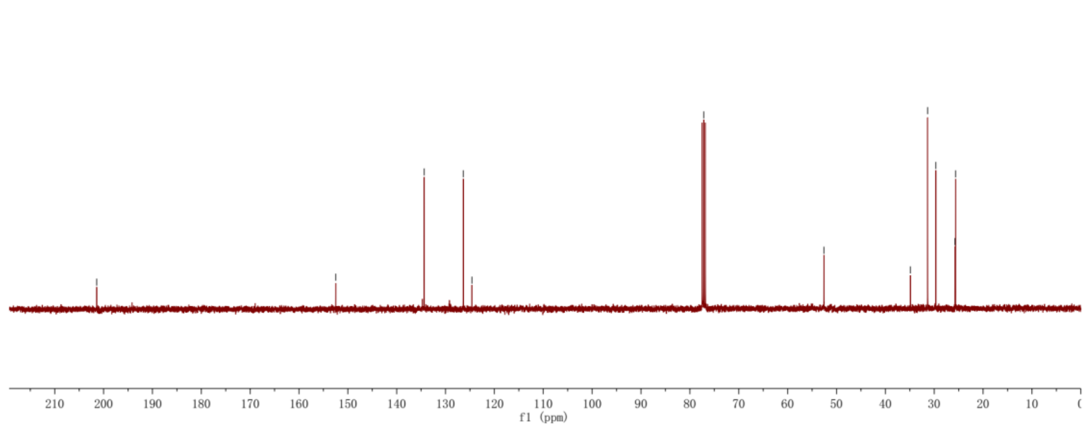

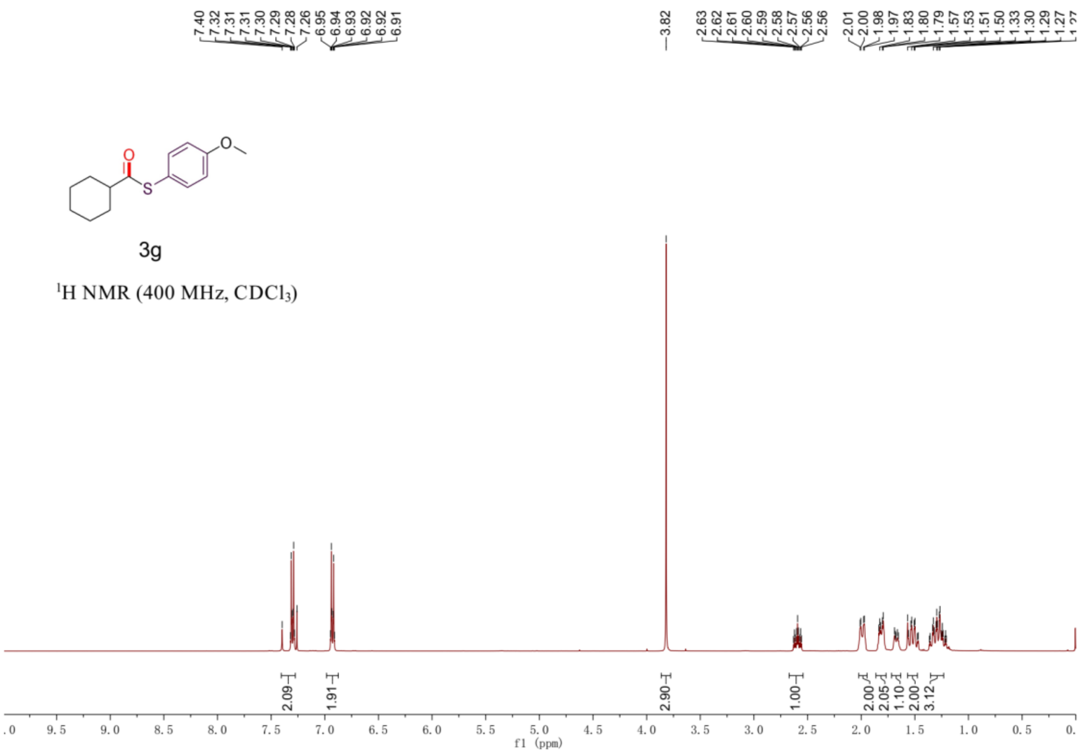
7.40
7.32
7.31
7.30
7.29
7.28
7.26
6.95
6.94
6.93
6.92
6.92
6.91
3.82
2.63
2.62
2.61
2.60
2.59
2.58
2.57
2.56
2.56
2.01
2.00
1.98
1.97
1.83
1.80
1.79
1.57
1.53
1.51
1.50
1.33
1.30
1.29
1.27
O
S
O
3g
1H NMR (400 MHz, CDCl3)
2.09
1.91
2.90
1.00
2.00
2.05
1.10
2.00
3.12
f1 (ppm)

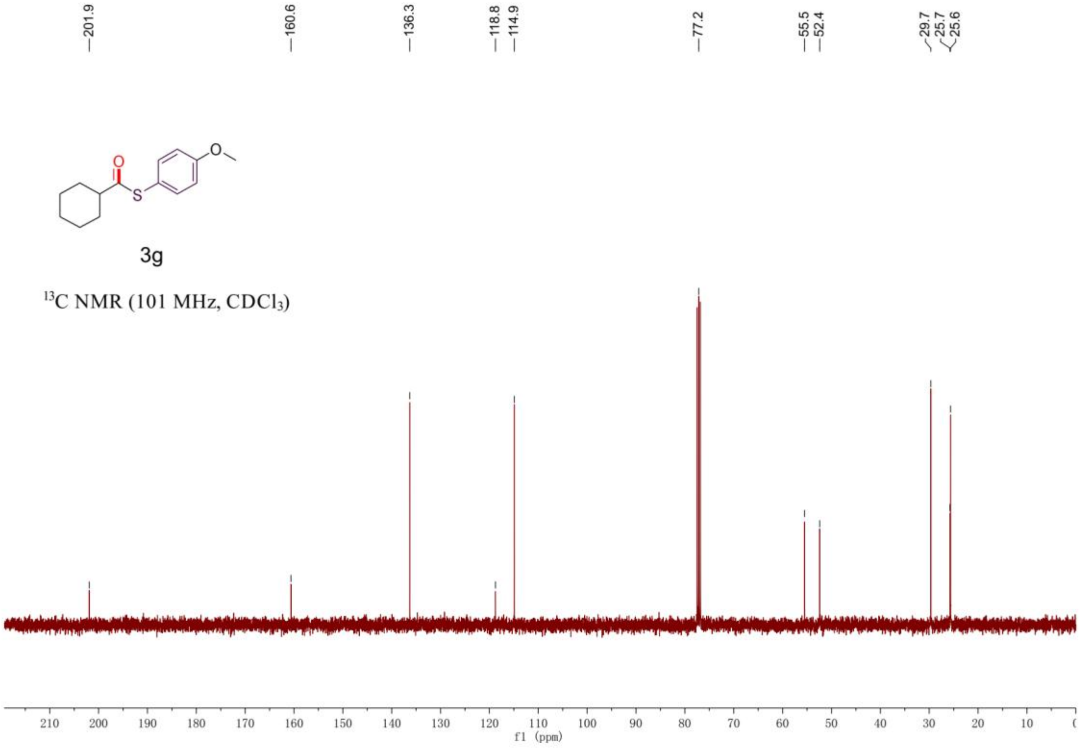
201.9
160.6
136.3
118.8
114.9
77.2
55.5
52.4
29.7
25.7
25.6
O
S
O
3g
13C NMR (101 MHz, CDCl3)
f1 (ppm)

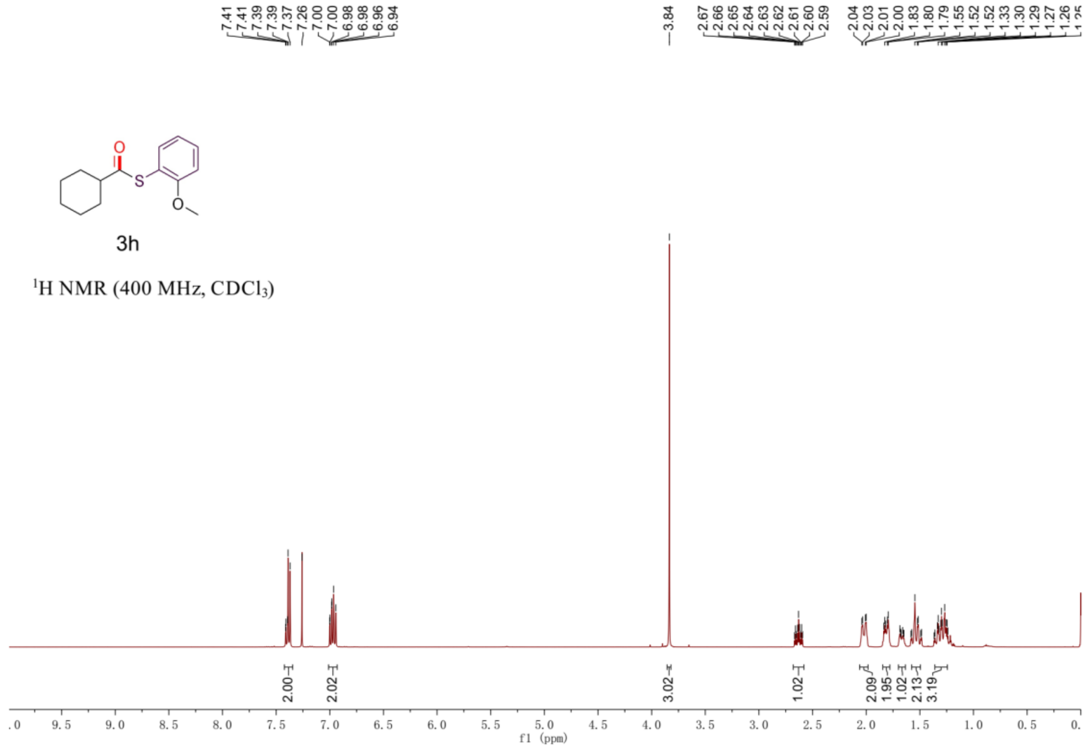
7.41 7.41 7.39 7.39 7.37 7.26 7.00 7.00 6.98 6.98 6.96 6.94
3.84
2.67 2.66 2.65 2.64 2.63 2.62 2.61 2.60 2.59
2.04 2.03 2.01 2.00 1.83 1.80 1.79 1.55 1.52 1.52 1.33 1.30 1.29 1.27 1.26
O
S
O
3h
^{1}H NMR (400 MHz, CDCl$_3$)
2.00
2.02
3.02
1.02
2.09
1.95
1.02
2.13
3.19
f1 (ppm)

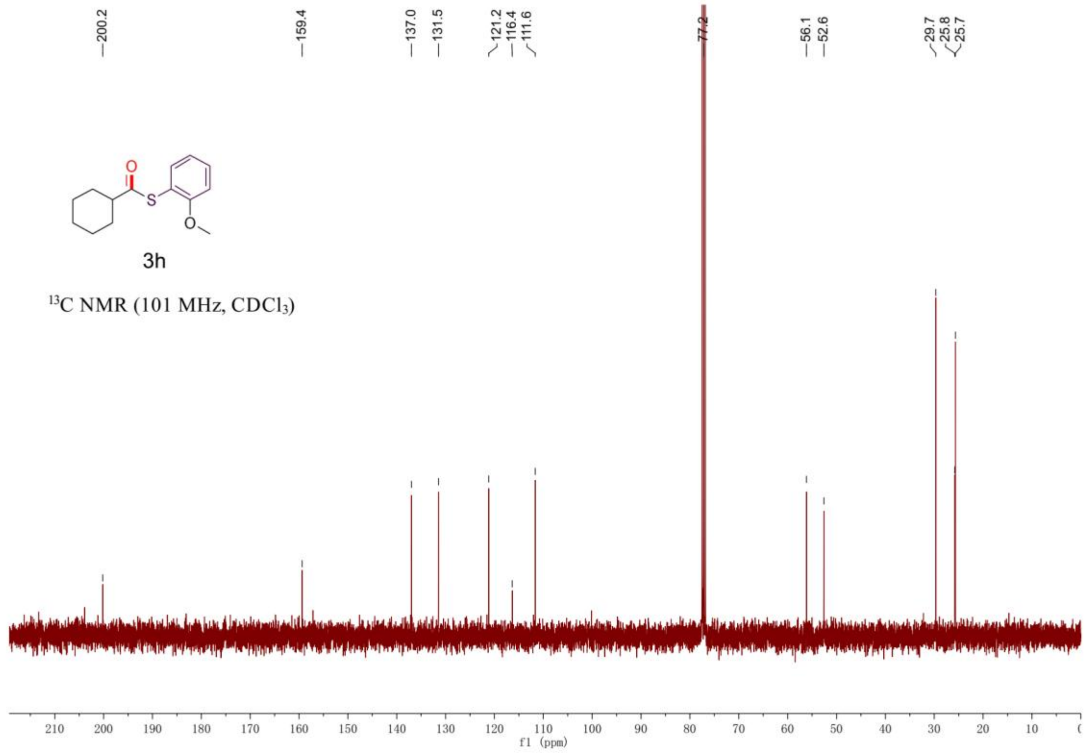
200.2
159.4
137.0
131.5
121.2
116.4
111.6
77.2
56.1
52.6
29.7
25.8
25.7
O
S
O
3h
^{13}C NMR (101 MHz, CDCl$_3$)
f1 (ppm)

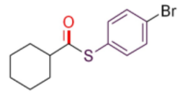

3i

^{1}H NMR (400 MHz, $CDCl_3$)

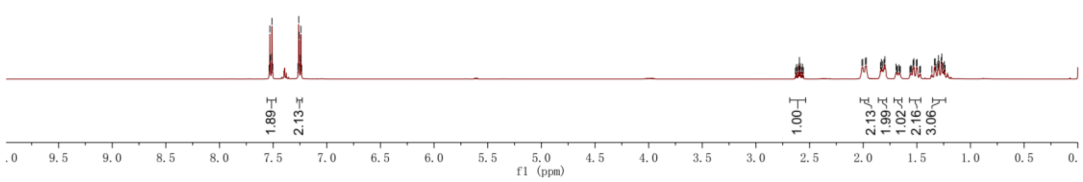

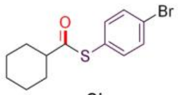

3i

^{13}C NMR (101 MHz, $CDCl_3$)

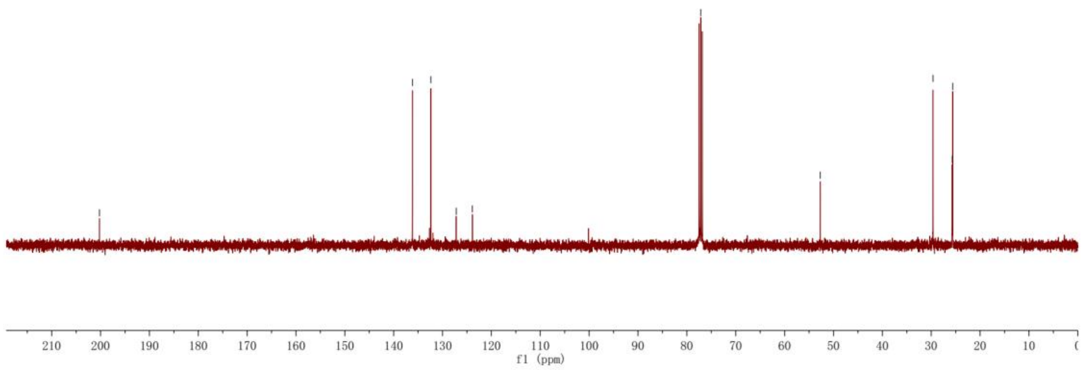

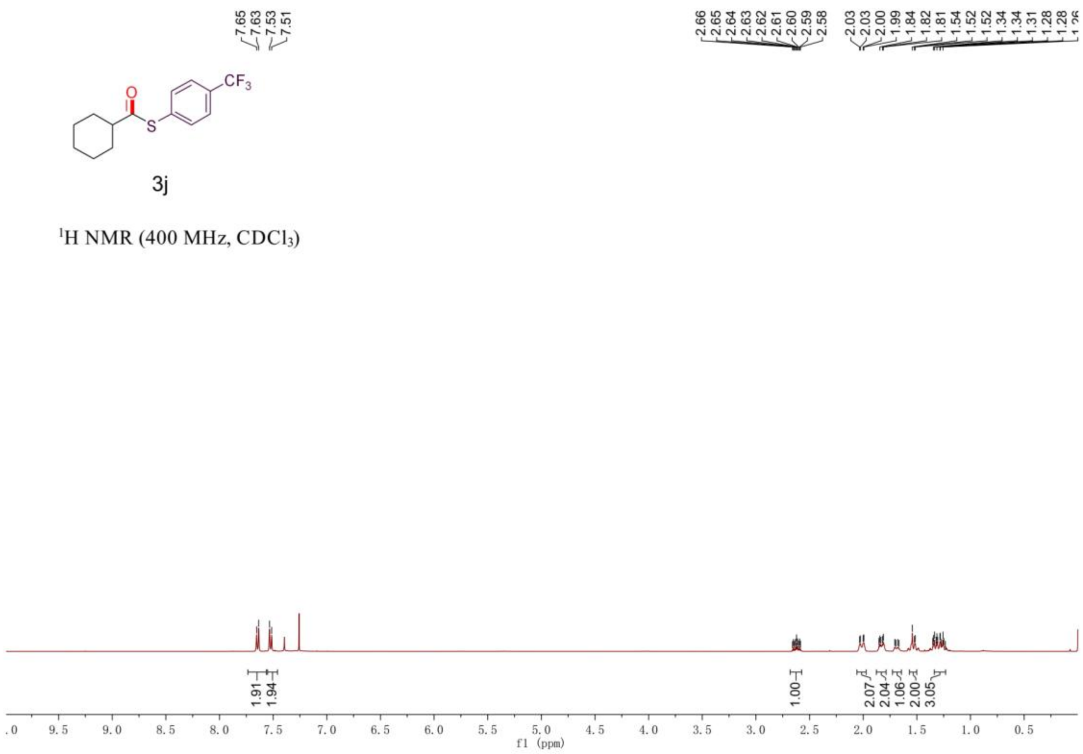
7.65
7.63
7.53
7.51
2.66
2.65
2.64
2.63
2.62
2.61
2.60
2.59
2.58
2.03
2.03
2.00
1.99
1.84
1.82
1.81
1.54
1.52
1.52
1.34
1.31
1.28
CF3
O
S
3j
1H NMR (400 MHz, CDCl3)
1.91
1.94
1.00
2.07
2.04
1.06
2.00
3.05
f1 (ppm)

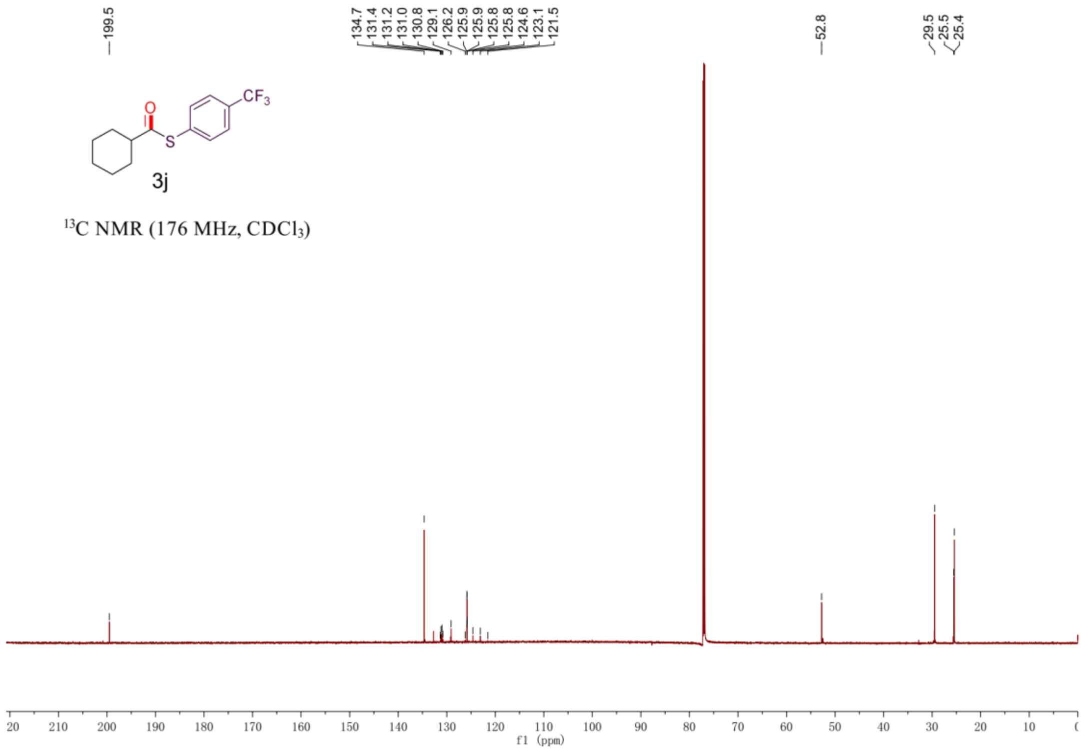
199.5
134.7
131.4
131.2
131.0
130.8
129.1
126.2
125.9
125.9
125.8
124.6
123.1
121.5
52.8
29.5
25.5
25.4
CF3
O
S
3j
13C NMR (176 MHz, CDCl3)
f1 (ppm)

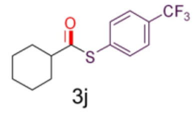

3j

^{19}F NMR (376 MHz, $CDCl_3$)

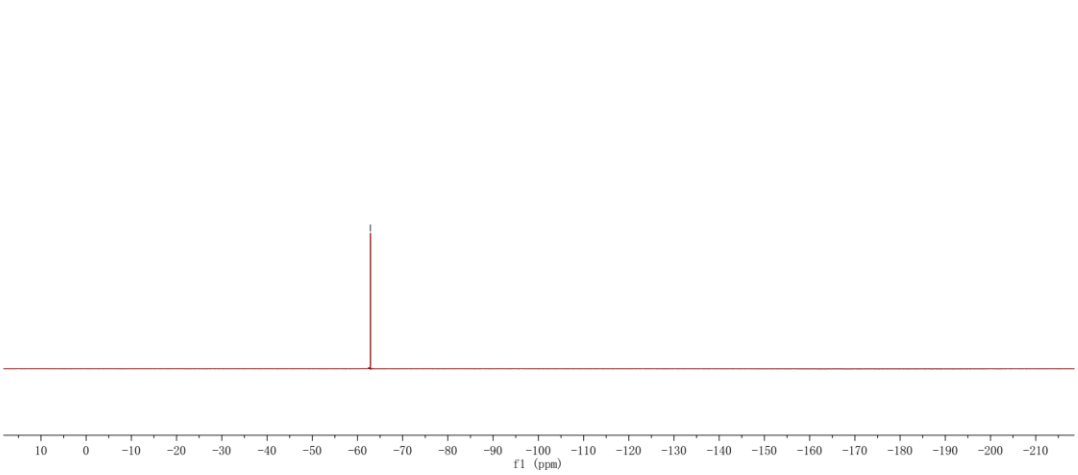

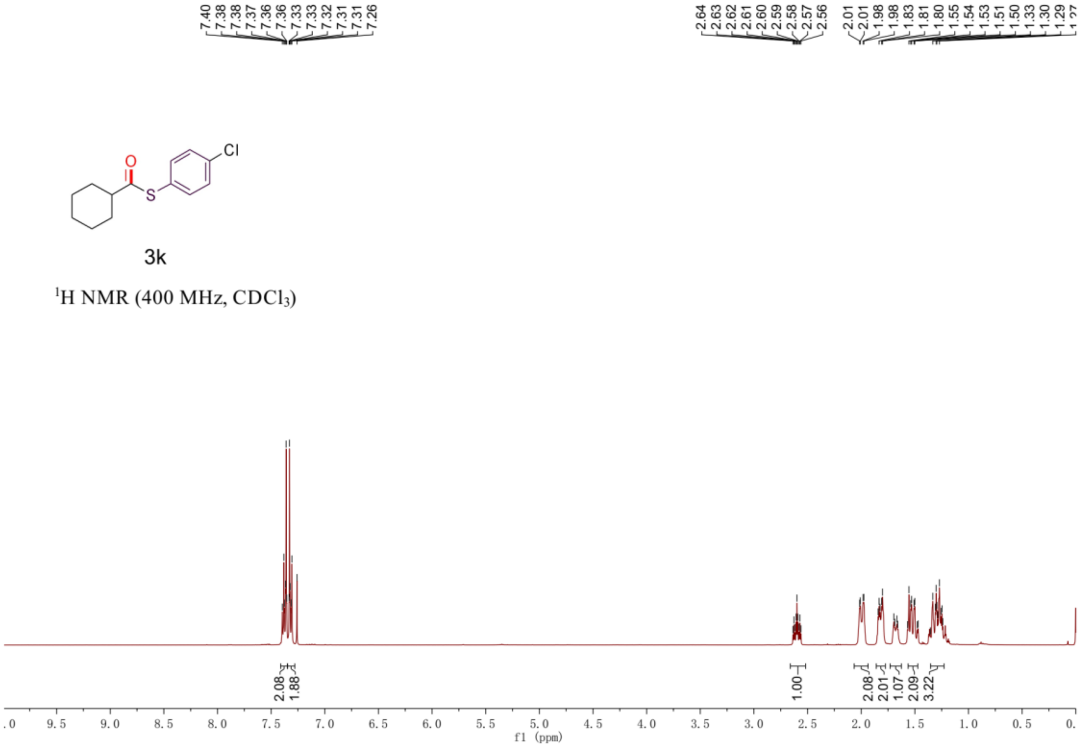

3k

^{1}H NMR (400 MHz, $CDCl_3$)

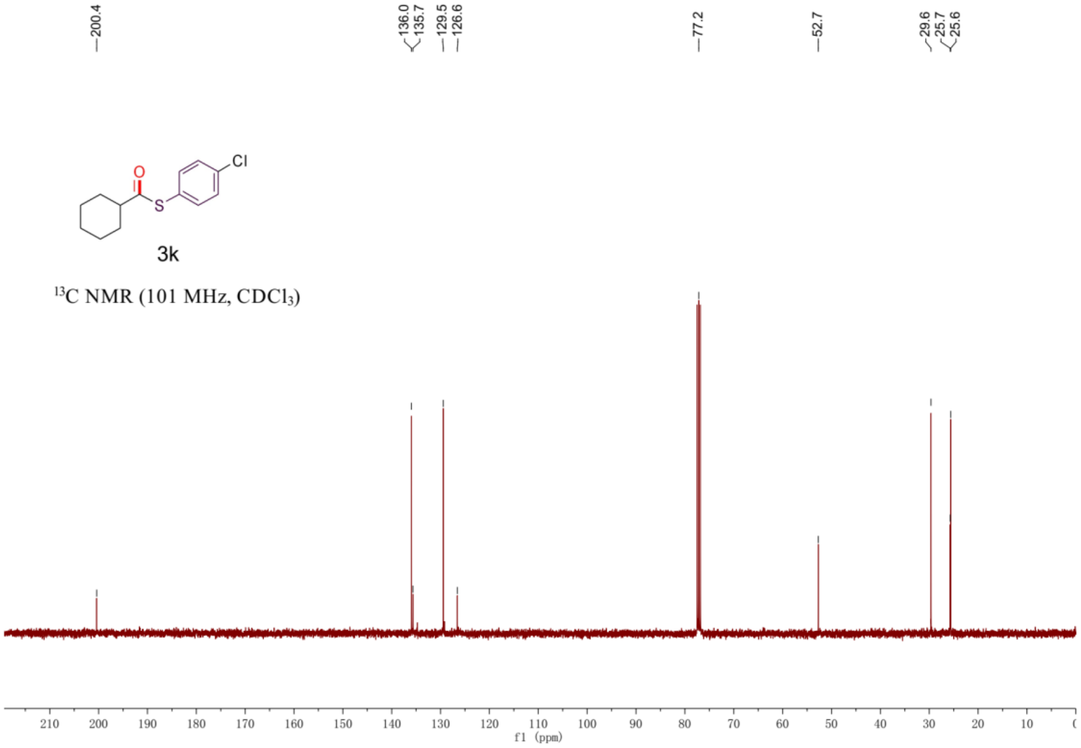

3k

^{13}C NMR (101 MHz, $CDCl_3$)

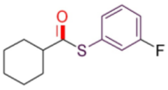

3l

^{1}H NMR (400 MHz, CDCl$_3$)

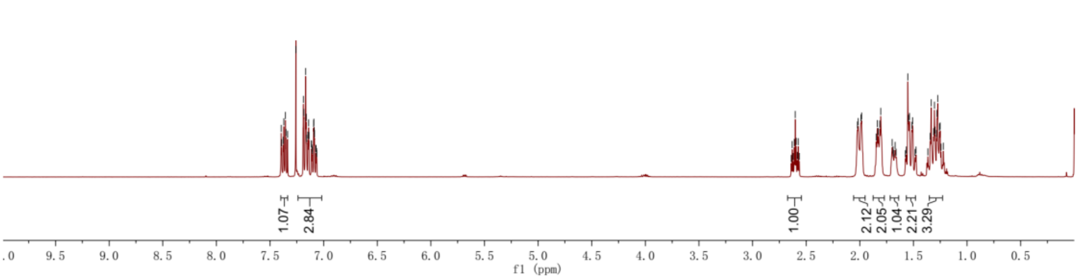

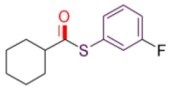

3l

^{13}C NMR (101 MHz, CDCl$_3$)

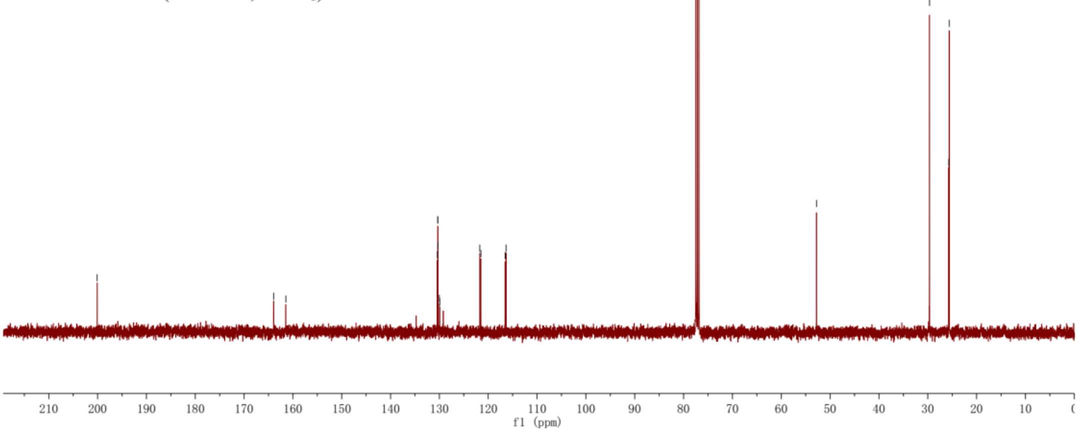

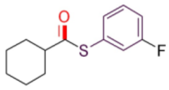

3l

^{19}F NMR (376 MHz, $CDCl_3$)

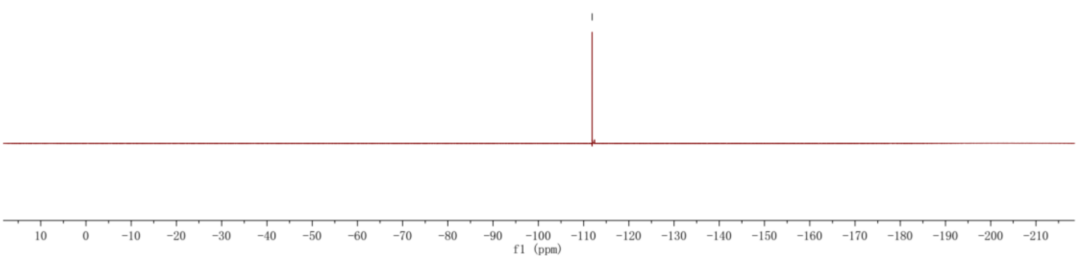

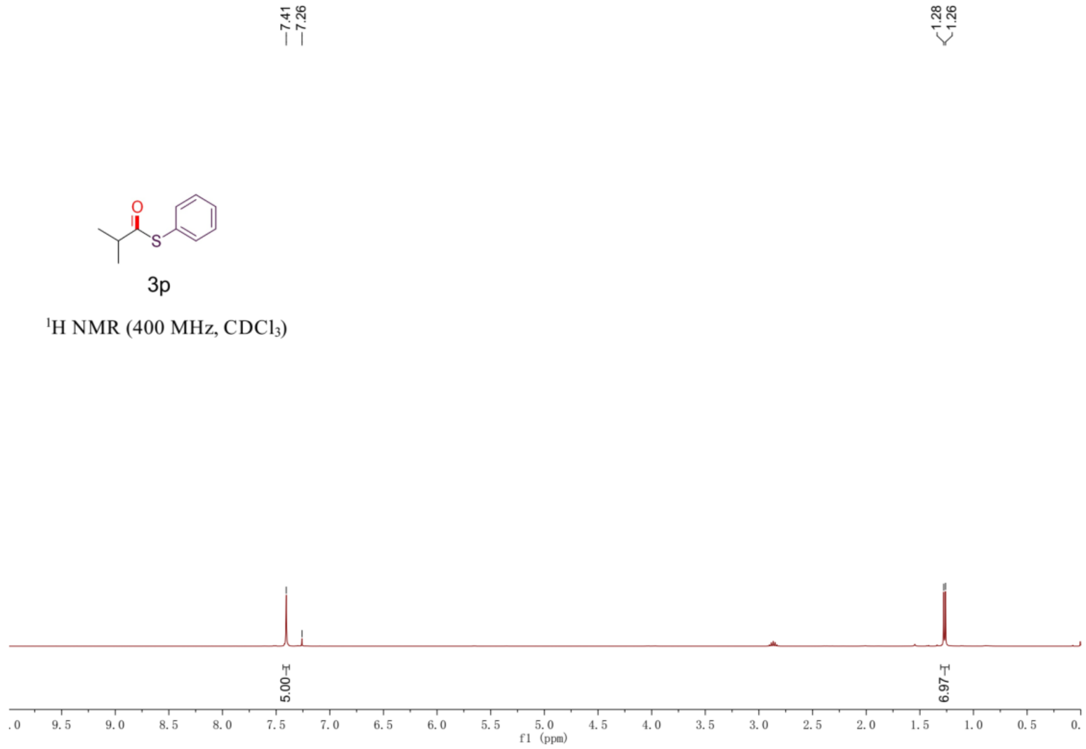
7.41
7.26
1.28
1.26
3p
1H NMR (400 MHz, CDCl3)
5.00
6.97
f1 (ppm)

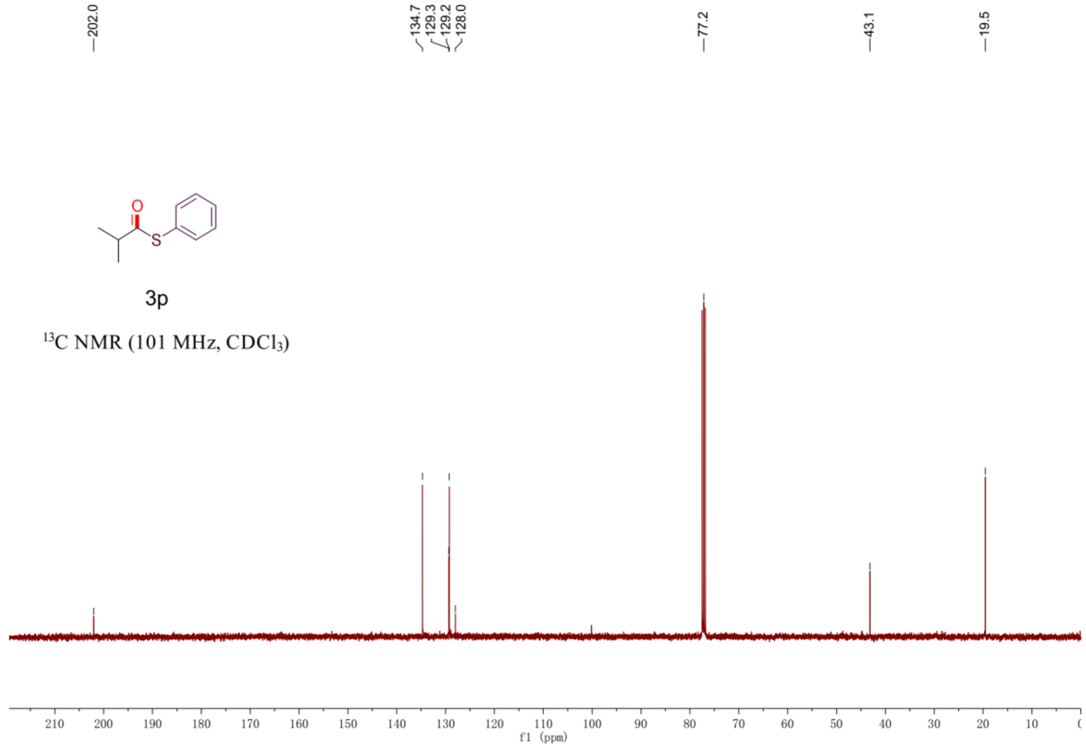
202.0
134.7
129.3
129.2
128.0
77.2
43.1
19.5
3p
13C NMR (101 MHz, CDCl3)
f1 (ppm)

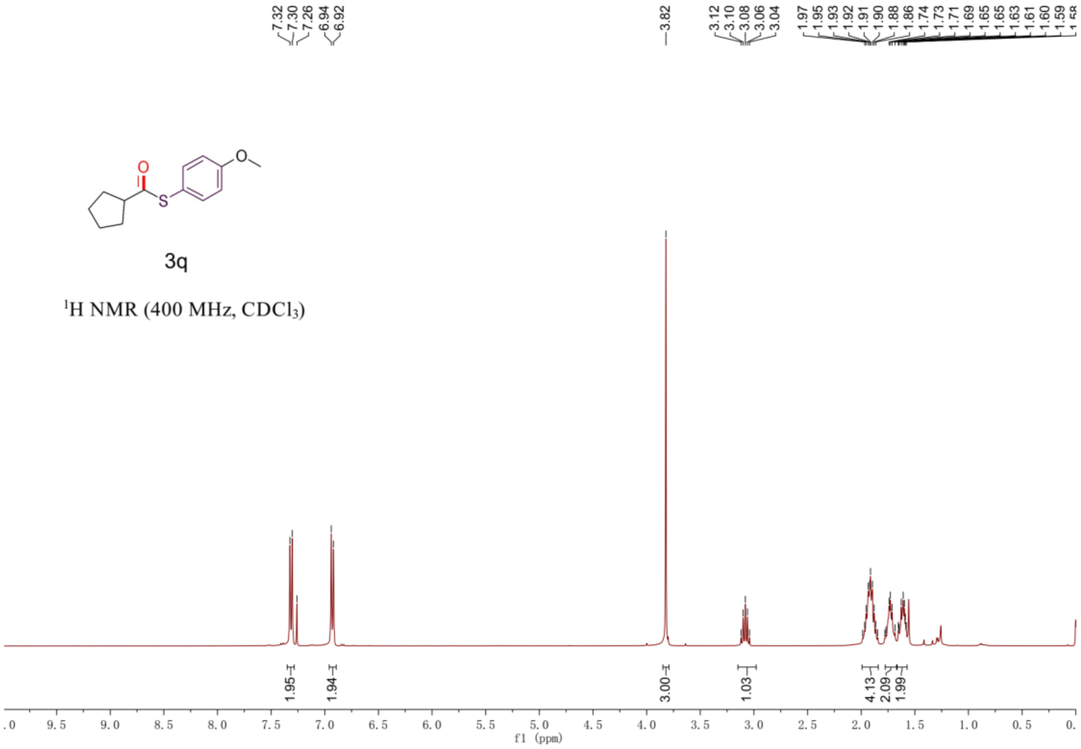
7.32
7.30
7.26
6.94
6.92
3.82
3.12
3.10
3.08
3.06
3.04
1.97
1.95
1.93
1.92
1.91
1.90
1.88
1.86
1.74
1.73
1.71
1.69
1.65
1.63
1.61
1.60
1.59
O
S
O
3q
¹H NMR (400 MHz, CDCl₃)
1.95
1.94
3.00
1.03
4.13
2.09
1.99
f1 (ppm)

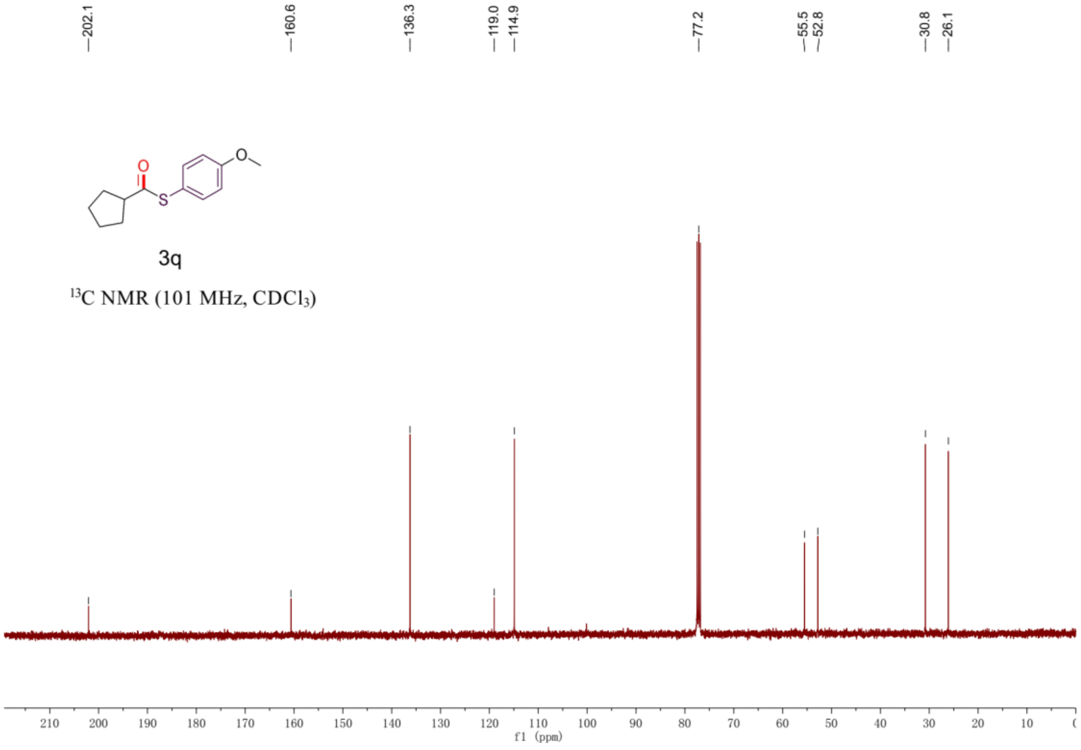
202.1
160.6
136.3
119.0
114.9
77.2
55.5
52.8
30.8
26.1
O
S
O
3q
¹³C NMR (101 MHz, CDCl₃)
f1 (ppm)

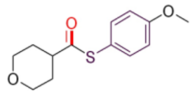

3r

^{1}H NMR (700 MHz, $CDCl_3$)

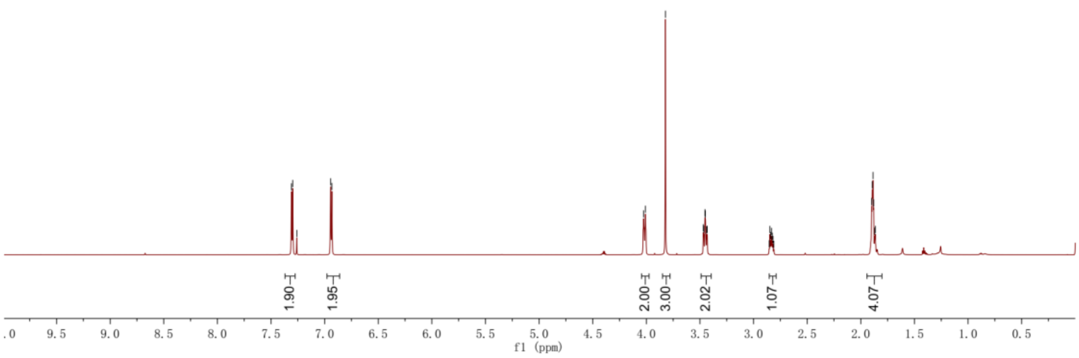

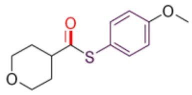

3r

^{13}C NMR (176 MHz, $CDCl_3$)

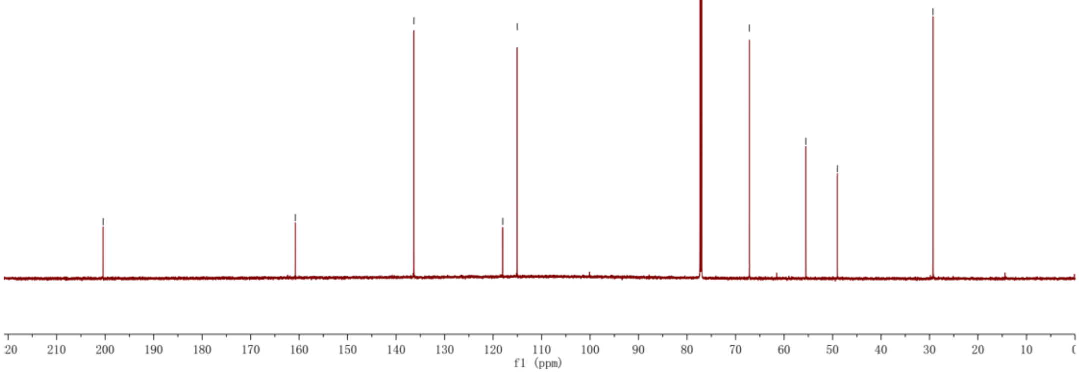

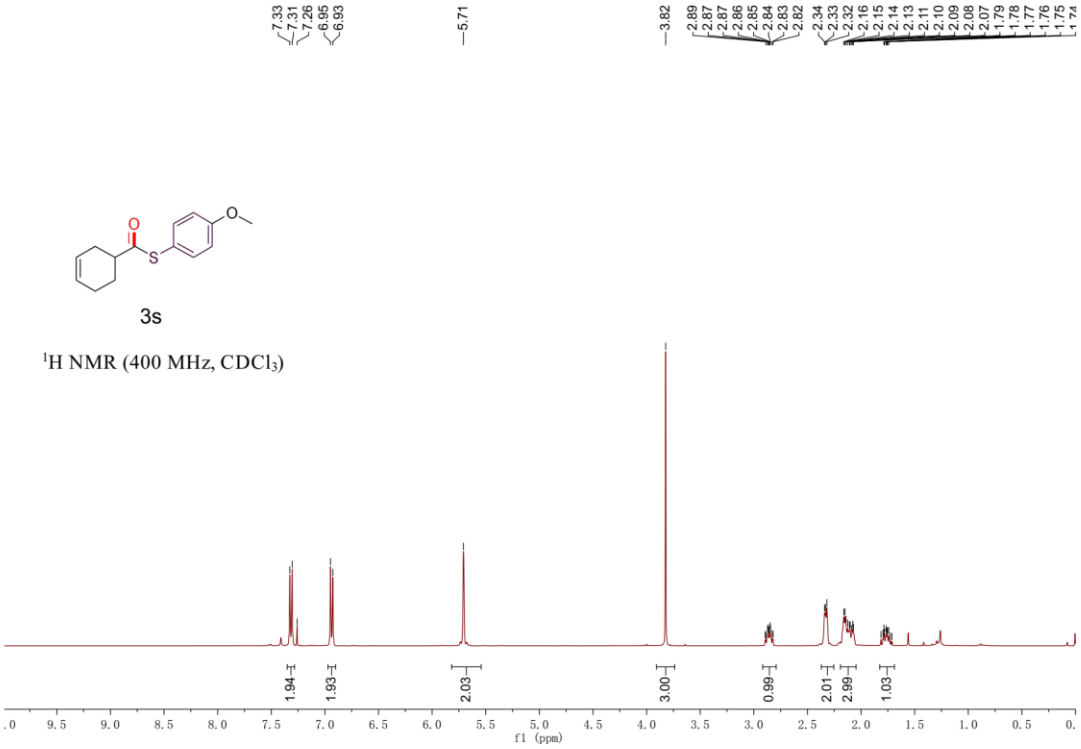
7.33
7.31
7.26
6.95
6.93
5.71
3.82
2.89
2.87
2.87
2.86
2.85
2.84
2.83
2.82
2.34
2.33
2.32
2.16
2.15
2.14
2.13
2.11
2.10
2.09
2.08
2.07
1.79
1.78
1.77
1.76
1.75
O
S
O
3s
1H NMR (400 MHz, CDCl3)
1.94
1.93
2.03
3.00
0.99
2.01
2.99
1.03
f1 (ppm)

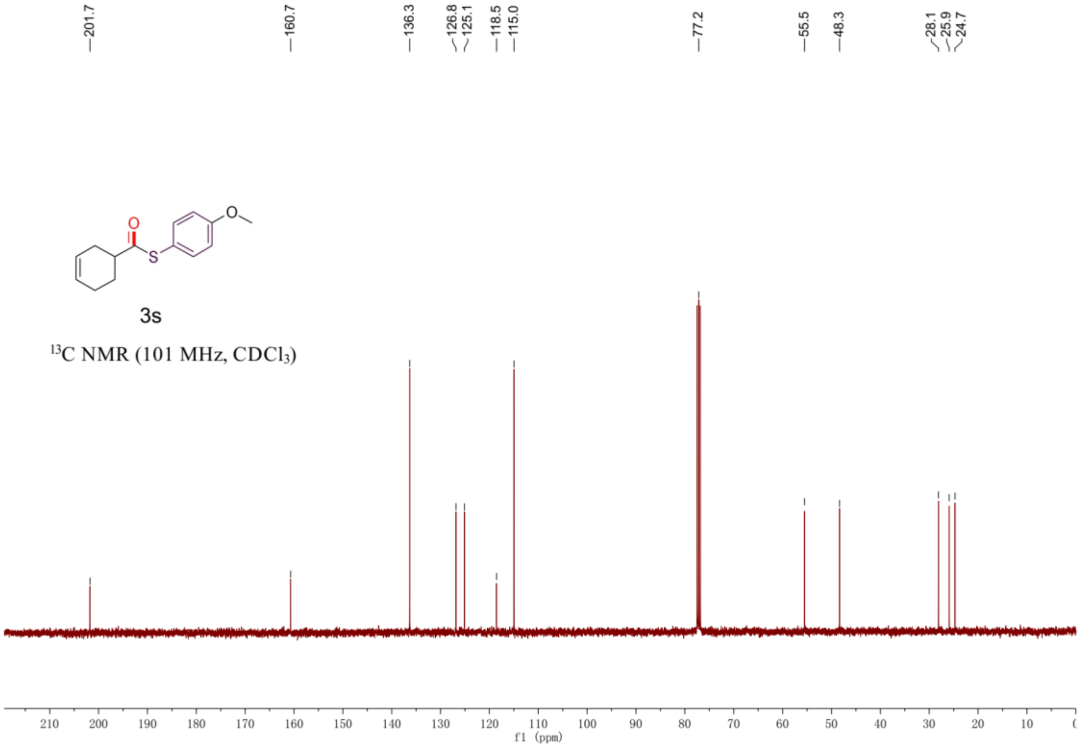
201.7
160.7
136.3
126.8
125.1
118.5
115.0
77.2
55.5
48.3
28.1
25.9
24.7
O
S
O
3s
13C NMR (101 MHz, CDCl3)
f1 (ppm)

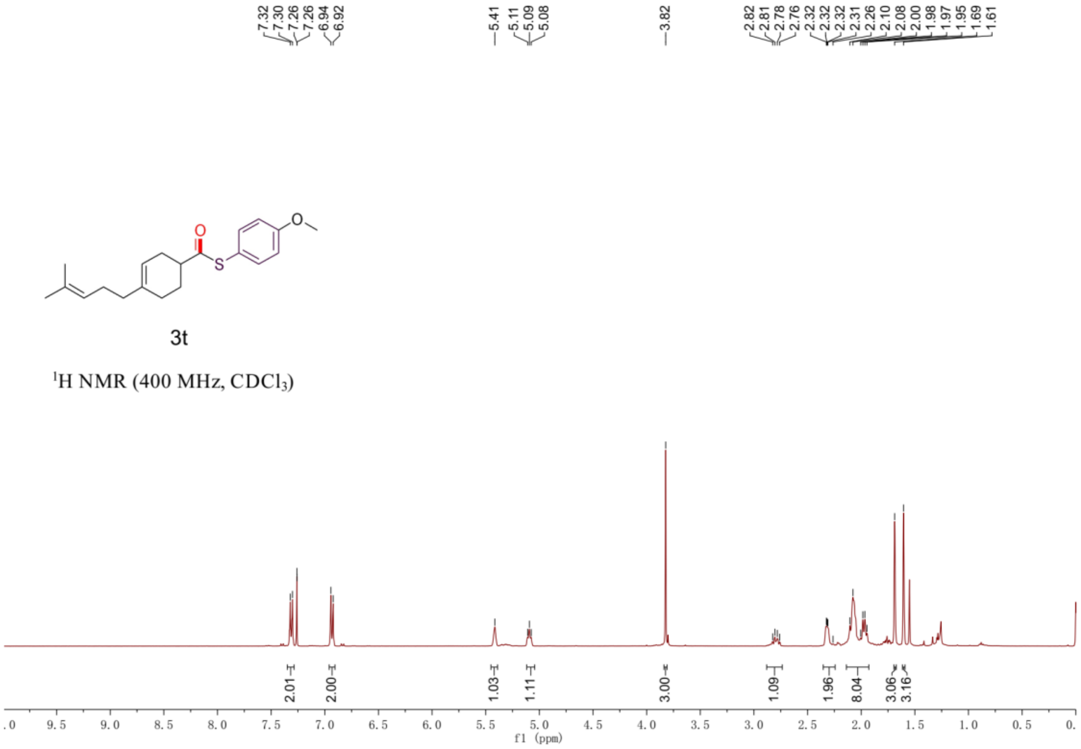

3t

^{1}H NMR (400 MHz, $CDCl_3$)

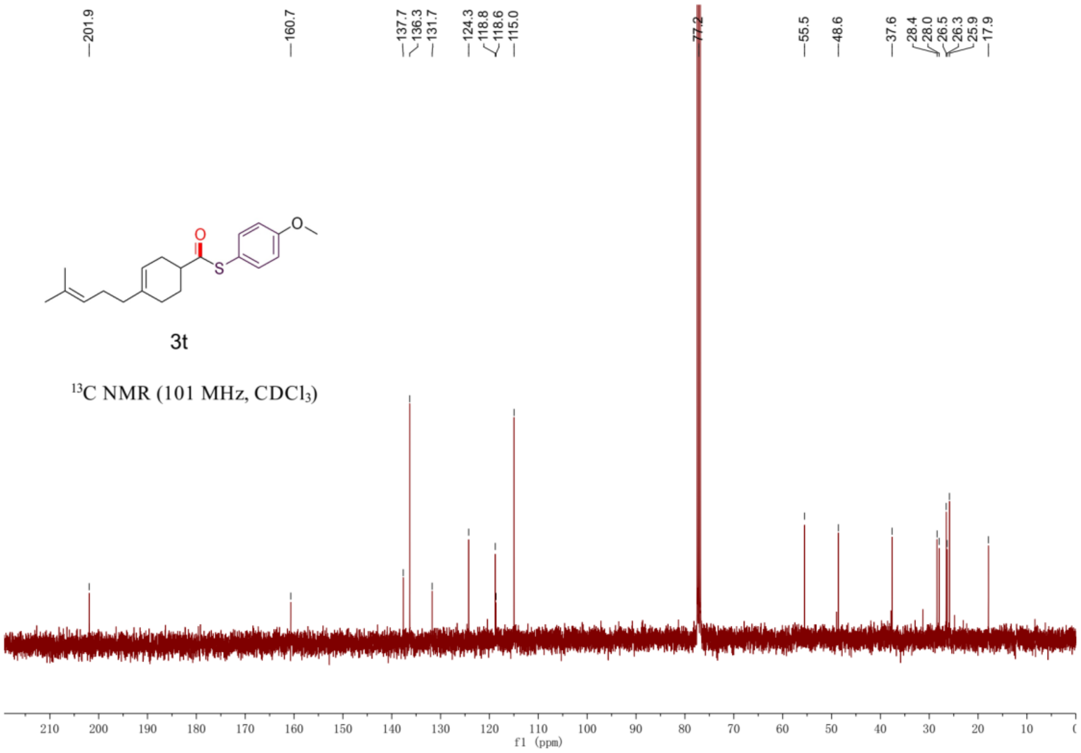

3t

^{13}C NMR (101 MHz, $CDCl_3$)

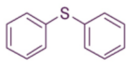

4a

^{1}H NMR (400 MHz, $CDCl_3$)

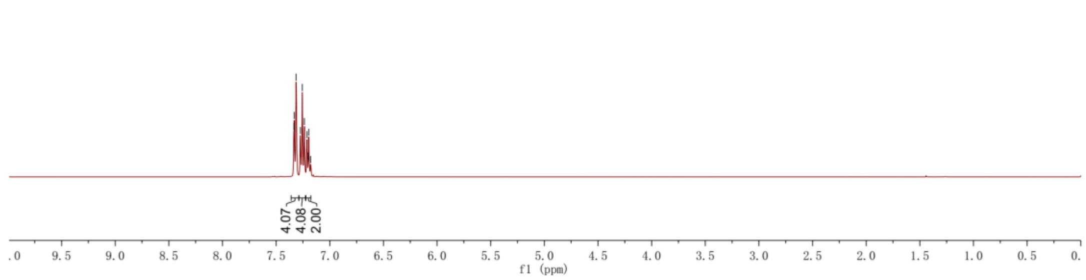

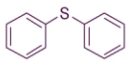

4a

^{13}C NMR (101 MHz, $CDCl_3$)

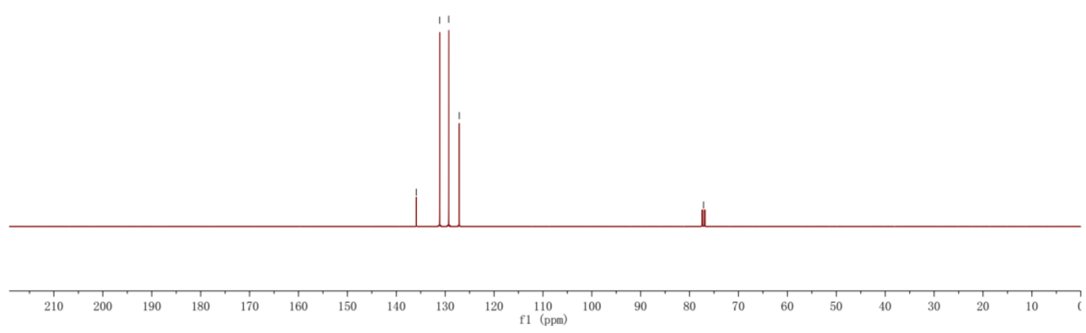

Supplement: Supplementary file 1 [file ol6c00753_si_001.pdf]
